# Exploiting Indian landraces to develop biofortified grain sorghum with high protein and minerals

**DOI:** 10.3389/fnut.2023.1228422

**Published:** 2023-10-09

**Authors:** Mallela Venkata Nagesh Kumar, Vittal Ramya, Setaboyine Maheshwaramma, Kuyyamudi Nanaiah Ganapathy, Mahalingam Govindaraj, Kosnam Kavitha, Kalisetti Vanisree

**Affiliations:** ^1^Professor Jayashankar Telangana State Agricultural University, Hyderabad, India; ^2^Indian Council of Agricultural Research-Indian Institute of Millets Research, Hyderabad, India; ^3^HarvestPlus Program, The Alliance of Bioversity International and the International Center for Tropical Agriculture (CIAT), Cali, Colombia

**Keywords:** sorghum, landraces, protein, biofortification, nutrition and health security

## Abstract

Sorghum (*Sorghum bicolor* L. Moench) is the staple cereal and is the primary source of protein for millions of people in Asia and sub-Saharan Africa. Sorghum grain value has been increasing in tropical countries including India owing to its gluten-free nature, anti-oxidant properties and low glycemic index. However, the nutrient composition of modern cultivars is declining thus necessitating genetic biofortification of sorghum to combat malnutrition and improve nutritional balance in the human diet. Keeping this in view, efforts were made to utilize valuable alleles, associated with nutrient composition, that might have been left behind in the varietal development in sorghum. The study aimed to determine the genetic improvement for nine nutritional and quality parameters (crude protein, *in vitro* protein digestibility (IVPD), total iron (Fe), total zinc (Zn), bioavailable Fe (%), bioavailable Zn (%), total phenolics, tannins and antioxidant activity) in the grains of 19 sorghum genotypes (high yield, drought and grain mold tolerant) developed from 11 superior India’s landraces. After selection and advancement made from 2017 to 2022 through single seed descent method, the improvement in the nine nutritional and quality parameters was assessed. Significant variation was observed for all the nine parameters among the landraces and the genotypes. Sorghum genotypes PYPS 2 and PYPS 13 recorded the highest crude protein (13.21 and 12.80% respectively) and IVPD (18.68 and 19.56% respectively). Majority of the sorghum genotypes recorded high Fe (14.21–28.41 mg/100 g) and Zn (4.81–8.16 mg/100 g). High phenolics and antioxidant activity were recorded in sorghum genotypes PYPS 18 (85.65 mg/g gallic acid equivalents) and PYPS 19 (89.78%) respectively. Selections through SSD method revealed highest improvement in genotype PYPS 10 for crude protein (32.25%), total phenolics (18.48%) and antioxidant activity (15.43%). High improvements in genotypes PYPS 12 (23.50%), PYPS 3 (26.79%), PYPS 15 (21.18%) were recorded for total Fe, available Fe and high tannins, respectively. The study demonstrated that landraces could be effectively utilized as a potential, low-cost and eco-friendly approach in sorghum genetic biofortification to improved sorghum productivity and nutritional supply in semi-arid tropics.

## Introduction

Sorghum (*Sorghum bicolor* L. Moench) is one of the world’s most important cereal crops, ranking fifth in terms of production after maize, rice, wheat and barley (1). Sorghum is cultivated in more than 100 countries, primarily in Asia and sub-Saharan Africa (2). In 2021, the world sorghum production was estimated to be of 60.10 million tonnes in an area of 45.90 million ha and productivity of 1,309 kg/ha (1). The top sorghum-producing countries in the world are United States of America, Nigeria, Mexico, India and Sudan.

Sorghum is considered a climate-resilient crop due to its ability to adapt to a variety of agro-ecological conditions, including drought-proneness, limited resources, and erratic rainfall patterns (3). Under harsh conditions, as a climate-smart crop, sorghum is helpful for small-holder farmers as it can help to ensure food security for millions of people who live in these regions.

Sorghum is a multi-purpose crop that is used for food, feed and fodder, making it an essential component of food systems in many developing countries, particularly in the Indian subcontinent and sub-Saharan Africa (4). From a nutritional point of view, it is an important dietary staple and a cheap source of energy for millions of people living in resource-poor areas. Sorghum is rich in protein, dietary fiber, and important micronutrients such as zinc and iron (5). It has gained popularity as a gluten-free crop with a low glycemic index and high antioxidant properties (6, 7). Sorghum is a good source of phenolic compounds including phenolic acids, flavonoids, anthocyanins, phytosterols, condensed tannins and carotenoids (lutein and zeaxanthin), thus making the grain suitable for developing functional foods and nutraceuticals (8, 9). Some potential health and pharmaceutical benefits of sorghum include slow digestibility, cholesterol-lowering, antioxidant, anti-inflammatory, and anti-carcinogenic properties (8, 10, 11). Sorghum is also an important feed crop for livestock, particularly in areas where the pastureland is limited (12).

In India, sorghum is an important crop grown on over 9 million ha with a production of over 11 million tonnes (1). It is particularly important in the dryland regions of India for the small-holder farmers and is often grown as a subsistence crop (13). However, sorghum cultivation faces various constraints including the prevalence of pests and diseases and limited availability of improved varieties to meet the demands of farmers and consumers (14). Due to limited access to high-yielding and disease-resistant sorghum many farmers are continuing to use traditional, low-yielding cultivars, which is constraining the sorghum production (15). There is a need for continuous development of improved varieties to address the challenges, including low yields, faced by sorghum farmers to enhance food security.

Landraces are traditional, locally adapted cultivars that have been developed by farmers through a process of natural selection over many generations for traits such as tolerance to environmental stresses, nutritional content, flavor etc. They represent a valuable source of genetic diversity that can be utilized for crop improvement (16). Landraces have played a significant role in the domestication of sorghum, which is believed to have originated in Africa more than 5,000 years ago (17). Sorghum landraces have been extensively used in sorghum breeding programs, and continue to be utilized to develop improved varieties that cater to the diverse requirements of farmers and consumers. The wide range of genetic diversity and adaptation to local environmental conditions, make them an important genetic resource for breeding improved and biofortified sorghum (16).

India has a rich diversity of sorghum landraces with variations based on grain color, including yellow, red, white and brown, among which, red sorghum landraces are most commonly cultivated, followed by white and brown sorghum landraces (18). They are reported to contain high levels of protein, dietary fiber, and essential micronutrients such as iron, zinc, and calcium, and also possess phenolic compounds and antioxidant properties, making them a valuable resource for enhancing human health and nutritional security (19).

Sorghum is an important source of minerals, vitamins and proteins for human and animal health. Knowledge of nutritional diversity would have a direct impact on the improvement of sorghum for quality breeding (20). The Indian sorghum landraces are a valuable source of genetic diversity for yield and other economic traits (21). The different colored grains of sorghum landraces in India indicate their high genetic diversity and might be valuable sources in breeding programs for selection of specific traits such as high yield, drought tolerance, disease and pest resistance and nutritional quality. Keeping this in view, the present study was taken up with the objectives (i) to utilize sorghum landraces to develop sorghum genotypes using hybridization and continuous selection for nutritional improvement; (ii) to evaluate the genetic variation in the grains of parent landraces and their evolved genotypes for nine nutritional parameters (crude protein, IVPD, total Fe, bioavailable Fe, total Zn, bioavailable Zn, total phenolics, tannins and antioxidant activity); (iii) to assess the nutritional improvement in the evolved genotypes due to continuous selection.

## Materials and methods

### Development of sorghum genotypes

A collection of 36 diverse landraces (PSLRC 1-PSLRC 36), distinct for various characters viz., maturity, grain type, tolerances to grain mold disease and terminal moisture stress, maintained at the Regional Agricultural Research Station (RARS), Palem, Telangana, India were used in the study (Supplementary Table S1).The breeding program was focused on developing sorghum genotypes for improved agronomic performance and grain mold disease resistance. Eleven agronomically superior and grain mold tolerant sorghum landraces distinct for various characters viz., maturity, grain type, tolerances to grain mold disease and terminal moisture stress, maintained at the Regional Agricultural Research Station (R.A.R.S.), Palem, Telangana, India were utilized in hybridization followed by a selection from 2010 to 2015 in rainy and post-rainy seasons at RARS, Palem. A minimum population of 250 plants was maintained in each F_2_ and subsequent generation. They were advanced to F_6_ generation by using the pedigree method of selection. The F_6_ progeny of individual cross combination was considered as a single-sorghum advanced genotype having a diverse genetic background for agronomic and grain characters, and grain mold tolerance. Nineteen superior advanced sorghum genotypes were identified and further evaluated for nutritional improvement by continuous selection using the single seed descent (SSD) method where one seed from each generation within a genotype was harvested and advanced through multiple generations. The experiment was laid out in a randomized block design with three replications from 2017 to 2022 at R.A.R.S, Palem, Telangana, India. Each sorghum genotype was planted on six rows of 5 m length plots by using between- and within-row spacing of 45 and 10 cm, respectively. Weeds, insect pests, and foliar disease were managed as recommended for the crop by using a combination of cultural and chemical practices.

### Nutritional composition analysis

The grains of 11 sorghum parent landraces and 19 sorghum genotypes (before and after SSD breeding) were analyzed for crude protein, IVPD, total Fe, total Zn, bioavailable Fe (%), bioavailable Zn (%), total phenolics, tannins and anti-oxidant activity. Whole grains were collected from the fields where they were grown and the grains were cleaned, milled and sieved through 0.4 mm sieve. The fine flour was packaged and kept at 4°C until use in the nutritional analysis.

The crude protein of the sorghum grains was determined using the Kjeldahl method following AOAC (22). The in vitroprotein digestibility (IVPD) was determined using the formula by Monjula & John (23): IVPD % = (digestible protein ÷ total protein) × 100.

Total Fe and total Zn were extracted from each sample after burning in a muffle furnace at 550°C and then dissolving in 5 N HCl. The Fe and Zn contents were determined using an atomic absorption spectrophotometer (24). The bioavailability of Fe and Zn was determined using 1 g of the sample extracted in 10 mL of 0.03 M HCl for 3 h at 37°C. The clear extract was oven-dried at 60°C and then acid-digested according to Chauhan and Mahajan (25). Bioavailability (%) of Fe and Zn was determined using the formula: (mineral extractable in 0.03 N HCl ÷ total mineral) × 100.

The total phenols were extracted using Talhaoui et al. (26). Sorghum flour was suspended in methanol at a ratio of 1:25 (w/v) at 25°C for 24 h. The extract was then collected and the process was repeated. The collected extract was then vacuum-dried using a rotary evaporator to determine the total phenolic content (27). From the extract, 20 μL of the solution (1,10, w/v) was mixed with 1.58 mL H_2_O and 100 μL of the Folin–Ciocalteu reagent. Then, 300 μL of Na_2_CO_3_ was added to the solution and kept at 20°C for 2 h. The absorbance was detected at 765 nm in contradiction to the blank solution. A calibration curve was constructed using different concentrations of gallic acid (GAE) (*R*^2^ = 0.9672). The tannin content was determined using the spectrophotometric vanillin-HCl method (28). Different concentrations of catechin were prepared according to the standard curve and the tannin results were expressed as catechin equivalents. The anti-oxidant activity of the sorghum sample extracts obtained using Talhaoui et al. (26) was determined using the 2,2-diphenyl-1-picrylhydrazyl (DPPH) scavenging assay (29). A mixture of 0.9 mL of 50 mM Tris–HCl buffer with a pH of 7.4 and 0.1 mL of extracts (or deionized H_2_O as the control) was kept at the ambient temperature for 30 min. The absorbance was detected at 517 nm, and DPPH scavenging was calculated as DPPH scavenging (%) = [(Absorbance control – Absorbance sample) ÷ Absorbance control] × 100.

### Statistical analysis

The analysis of variance for each trait was carried out as per the standard statistical procedure described by Panse and Sukhatme (30) to test the differences between the genotypes for all the characters. The mean values of all the grain quality parameters were compared using LSD test at *p* < 0.05. A combined analysis of variance over the years for each character was also used for the effects of selection over years in various genotypes. Principal component analysis (PCA) was performed as an ordination method to visualize the relationships among the 9 nutrient parameters by landraces and genotypes and the biplots of the first two principal components were displayed for interpretation. PCA analysis was done in R cluster package (31). Pearson correlation coefficients between the grain quality traits were analysed using R studio software version 3.2.2 (32).

## Results

Nine nutrient and quality parameters of sorghum grains were estimated in 11 parent landraces and 19 genotypes evolved from these landraces before and after advancement using the SSD method (Supplementary Tables S2–S10). Further, the variability and correlation among these nine parameters were estimated in landraces and their genotypes.

### Nutritional composition in sorghum landraces

#### Crude protein and IVPD

The crude protein content of the sorghum landraces exhibited significant (*p* < 0.05) variation and ranged from 8.51 to 11.82% (Table 1). Landraces PSLRC 2, PSLRC 7, PSLRC 20 and PSLRC 21 exhibited significantly (*p* < 0.05) higher crude protein contents compared to the other sorghum landraces. PSLRC-10 and 12 had significantly lower crude protein contents than the other sorghum landraces (*p* < 0.05). There were no significant differences in the crude protein contents of landraces PSLRC 4, PSLRC 8 and PSLRC 9 (Table 1). There was significant variation (*p* < 0.05) in the IVPD of the 11 sorghum landraces, which ranged from 11.64 to 15.81% (Table 1). Landraces PSLRC 3, PSLRC 7 and PSLRC 21 had significantly (*p* < 0.05) higher IVPD contents of 15.81, 15.48 and 14.81%, respectively, and landrace PSLRC 9 had significantly (*p* < 0.05) low IVPD content (11.64%).

**Table 1:** Mean performance of grain nutrients and quality parameters of sorghum landraces during 2011.

S. No.	Land race	Crude protein (%)	IVPD (%)	Fe (mg/100 g)	Available Fe (%)	Zn (mg/100 g)	Available Zn (%)	Phenolics (mg/g GAE)	Tannins (mg/g)	Anti-oxidant activity (%)
1	PSLRC-2	11.35^abc^	14.52^b^	15.82^ab^	38.32^i^	4.72^f^	48.62^c^	56.52^a^	24.82^d^	70.32^a^
2	PSLRC-3	10.52^bcd^	15.81^a^	12.51^f^	37.61^i^	5.21^cdef^	41.31^e^	51.31^b^	23.61^e^	
3	PSLRC-4	9.58^de^	12.70^c^	14.95^bc^	42.35^h^	4.85^ef^	46.75^d^	50.05^c^	20.25^f^	43.55^h^
4	PSLRC-6	10.38^cd^	13.55^c^	16.65^a^	51.55^d^	5.35^bcdef^	41.95^e^	45.75^f^	30.25^a^	66.85^c^
5	PSLRC-7	10.98^abc^	15.48^ab^	14.48^cd^	46.68^g^	5.88^abcde^	56.28^a^	43.58^g^	23.58^e^	45.28^g^
6	PSLRC-8	9.70^de^	12.70^c^	16.20^a^	55.60^b^	6.20^abc^	50.80^b^	49.70^cd^	29.40^a^	66.8^c^
7	PSLRC-9	9.54^de^	11.64^d^	13.84^de^	59.34^a^	6.84^a^	51.74^b^	48.74^d^	26.34^c^	43.44^h^
8	PSLRC-10	8.51^f^	12.66^c^	16.46^a^	50.46^e^	5.06^def^	55.86^a^	46.26^f^	27.76^b^	48.56^f^
9	PSLRC-12	9.02^ef^	13.35^c^	12.92^ef^	49.32^f^	5.42^bcdef^	56.32^a^	47.72^e^	20.32^f^	51.72^e^
10	PSLRC-20	11.52^ab^	13.52^c^	12.52^f^	51.82^d^	6.32^ab^	46.32^d^	50.32^c^	29.52^a^	68.32^b^
11	PSLRC-21	11.82^a^	14.81^ab^	12.01^f^	54.32^c^	6.01^abcd^	48.21^c^	51.82^b^	28.31^b^	63.51^d^
	CD:	0.95	0.95	0.95	0.95	0.95	0.95	0.95	0.95	0.94
	CV:	5.40	4.04	3.85	1.13	9.85	1.12	1.13	1.13	0.96

IVPD, In vitro protein digestibility. Means followed by a common letter are not significantly different at 5% level.

#### Total and available Fe and Zn

The total and available Fe and Zn varied among the landraces. Significant variation was observed in the total Fe content (*p* < 0.05) ranging from 2.01 mg/100 g to 16.65 mg/100 g (Table 1). PSLRC 2, PSLRC 6, PSLRC 8 and 10 had significantly (*p* < 0.05) higher total Fe and landraces PSLRC 3, PSLRC 12, PSLRC 20 and PSLRC 21 had significantly (*p* < 0.05) lower values (Table 1). The available Fe showed significant (*p* < 0.05) variation among the landraces ranging from 37.61% (PSLRC 3) to 59.34% (PSLRC 9). All the landraces varied significantly in their available Fe content, except for landraces PSLRC 6 (51.55%) and PSLRC 20 (51.82%) and the landraces PSLRC 2 (38.32%) and PSLRC 3 (37.61). The landraces showed significant (*p* < 0.05) variation in their total Zn and available Zn values. The Zn content ranged from 4.72 mg/100 g (PSLRC 2) to 6.84 1 mg/100 g (PSLRC 9) and the available Zn ranged from 41.31% (PSLRC 3) to 56.32% (PSLRC 12; Table 1).

#### Total phenolics, tannins and antioxidant activity

The total phenolic content showed significant (*p* < 0.05) variation ranging from 43.58 mg/g GAE (PSLRC-7) to 56.52 mg/g GAE (PSLRC-2; Table 1). Significant (*p* < 0.05) variations were also noted in the tannins and anti-oxidant activity of the landraces. PSLRC-6 (30.25 mg/g), PSLRC-8 (29.40 mg/g) and PSLRC-20 (29.52 mg/g) showed significantly (*p* < 0.05) higher tannins and PSLRC-8 (20.25 mg/g) and PSLRC-12 (20.32 mg/g) showed significantly (*p* < 0.05) lower tannin content (Table 1). The anti-oxidant activity among the 11 sorghum landraces ranged from 43.55 (PSLRC-4) to PSLRC-2 (70.32%). Landraces PSLRC-3 (67.51%) and PSLRC-8 (66.8%) also showed high anti-oxidant activity (Table 1).

### Nutrient and quality parameters in sorghum genotypes evolved from landraces

#### Crude protein and IVPD

The crude protein showed significant (*p* < 0.05) variation in the 19 sorghum genotypes during 2022 ranging from 8.79 to 13.21% (Table 2). Yellow pericarp sorghum genotype PYPS-2 (13.21%) and PYPS-13 (12.80%) had the highest protein followed by PYPS 1 (11.85%), PYPS 15 (11.76%) and PYPS 5 (11.73%). Sorghum genotypes PYPS 8 (8.94%), PYPS 10 (9.35%) and PYPS 12 (9.42%) showed significantly (*p* < 0.05) lower protein contents (Table 2). Sorghum genotype PYPS 13 (19.56%) showed significantly (*p* < 0.05) higher IVPD followed by PYPS 2 (18.68%) and PYPS 15 (17.54%) (Table 2). Genotype PYPS 6 (12.55%) showed significantly (*p* < 0.05) lower IVPD followed by PYPS 11 (12.93%), PYPS 12 (13.36%) and PYPS 10 (13.38%; Table 2).

**Table 2:** Improvement of crude protein and *in vitro* protein digestibility (IVPD) in sorghum grains developed by single seed descent method during 2017–2022.

S. No.	Genotype	Pedigree	Developed during 2022		% improvement due to selection	
			Crude protein (%)	IVPD (%)	Crude protein	IVPD
1.	PYPS-1	PSLRC 2 × PSLRC 3	11.85^ab^	16.59^de^	7.24	19.87
2.	PYPS-2	PSLRC 2 × PSLRC 4	13.21^a^	18.68^b^	10.45	−5.75
3.	PYPS-3	PSLRC 3 × PSLRC 4	10.44^bcde^	13.72^gh^	27.01	13.01
4.	PYPS-4	PSLRC 2 × PSLRC 6	10.26^bcde^	15.87^ef^	13.37	10.52
5.	PYPS-5	PSLRC 21 × PSLRC 7	11.73^ab^	13.86^gh^	14.33	19.69
6.	PYPS-6	PSLRC 3 × PSLRC 6	10.48^bcde^	12.55^i^	31.66	19.87
7.	PYPS-7	PSLRC 3 × PSLRC 7	9.67^de^	15.44^f^	19.53	10.36
8.	PYPS-8	PSLRC 8 × PSLRC 9	8.94^e^	14.32^g^	19.52	7.83
9.	PYPS-9	PSLRC 8 × PSLRC 10	9.59^de^	15.77^ef^	25.03	8.24
10.	PYPS-10	PSLRC 9 × PSLRC 10	9.35^e^	13.38^ghi^	32.25	8.96
11.	PYPS-11	PSLRC 4 × PSLRC 6	8.79^e^	12.93^hi^	5.65	15.86
12.	PYPS-12	PSLRC 4 × PSLRC 12	9.42^e^	13.36^ghi^	1.84	6.03
13.	PYPS-13	PSLRC 2 × PSLRC 21	12.80^a^	19.56^a^	14.29	16.71
14.	PYPS-14	PSLRC 20 × PSLRC 21	11.67^abc^	16.52^de^	15.32	13.62
15.	PYPS-15	PSLRC 2 × PSLRC 7	11.76^ab^	17.54^c^	16.21	15.24
16.	PYPS-16	PSLRC 20 × PSLRC 7	10.60^bcde^	15.66^ef^	13.13	19.27
17.	PYPS-17	PSLRC 2 × PSLRC 8	11.39^abcd^	16.34^def^	11.23	14.11
18.	PYPS-18	PSLRC 3 × PSLRC 8	9.52^de^	16.85^cd^	19.00	22.90
19.	PYPS-19	PSLRC 4 × PSLRC 8	9.68^cde^	15.85^ef^	17.33	19.35
	CD		0.97	0.84		
	CV		5.55	3.28		
	General Mean:		10.59	15.52		

Means followed by a common letter are not significantly different at 5% level.

#### Total and available Fe and Zn

The genotypes showed significant (*p* < 0.05) variation in their total (mg/100 g) and bioavailable Fe in the sorghum grains (Table 3). Genotype PYPS 11 (28.41 mg/100 g) had significantly (*p* < 0.05) higher total Fe content followed by PYPS 12 (26.80 mg/100 g) and PYPS 8 (26.06 mg/100 g). Interestingly PYPS 2 (14.21 mg/100 g) showed significantly (*p* < 0.05) lower total Fe content followed by genotypes PYPS 4 (15.35 mg/100 g) and PYPS 5 (15.48; Table 3). The available (%) Fe was significantly (*p* < 0.05) high in sorghum genotype PYPS 17 (73.22%) followed by PYPS 4 (72.65%) and PYPS 15 (72.14%) (Table 3). Genotype PYPS 12 (44.80%) showed significantly (p < 0.05) lower available Fe followed by the genotype PYPS 11 (48.32%). Similar to the Fe, the total and bioavailable Zn contents varied significantly (*p* < 0.05) among the sorghum genotypes with the highest total and bioavailable Zn recorded in PYPS 2 (8.16 mg/100 g) and PYPS 3 (72.76%) respectively (Table 3). Lower total Zn and bioavailable Zn contents were found in PYPS 11 (4.81 mg/100 g) and PYPS 8 (44.46%) respectively (Table 3).

**Table 3:** Improvement of total iron, total zinc, bioavailable iron and bioavailable zinc in sorghum developed by single seed descent method during 2017–2022.

S. No.	Genotype	Pedigree	Developed during 2022			% Improvement due to selection				
			Total iron (mg/100 g)	Total zinc (mg/100 g)	Bioavailable iron (%)	Bioavailable zinc (%)	Total iron	Total zinc	Bioavailable iron	Bioavailable zinc
1.	PYPS-1	PSLRC 2 × PSLRC 3	18.62^gh^	7.83^ab^	48.73^j^	52.93^i^	8.13	32.04	14.31	3.12
2.	PYPS-2	PSLRC 2 × PSLRC 4	14.21^i^	8.16^a^	54.16^g^	49.36^m^	10.93	−3.55	11.07	12.80
3.	PYPS-3	PSLRC 3 × PSLRC 4	20.55^ef^	5.66^cdef^	50.16^i^	72.76^a^	16.43	18.91	26.79	12.88
4.	PYPS-4	PSLRC 2 × PSLRC 6	15.35^i^	7.45^abc^	72.65^ab^	56.45^h^	12.45	−2.61	6.29	11.67
5.	PYPS-5	PSLRC 21 × PSLRC 7	15.48^i^	5.18^ef^	58.28^e^	50.18^lm^	4.74	8.37	18.02	11.07
6.	PYPS-6	PSLRC 3 × PSLRC 6	19.20^fg^	5.90^bdef^	61.10^d^	48.00^n^	−2.54	−7.81	9.69	10.85
7.	PYPS-7	PSLRC 3 × PSLRC 7	23.24^c^	5.34^ef^	64.44^c^	60.34^f^	−5.68	10.33	5.05	17.30
8.	PYPS-8	PSLRC 8 × PSLRC 9	26.06^b^	7.46^abc^	52.76^h^	44.46^p^	14.50	−5.09	13.56	6.47
9.	PYPS-9	PSLRC 8 × PSLRC 10	19.52^efg^	6.82^abcde^	56.42^f^	65.62^d^	10.78	−5.54	10.15	6.15
10.	PYPS-10	PSLRC 9 × PSLRC 10	22.82^cd^	5.42^def^	60.32^d^	62.32^e^	−5.78	25.46	7.10	12.45
11.	PYPS-11	PSLRC 4 × PSLRC 6	28.41^a^	4.81^f^	48.32^j^	69.21^b^	5.97	14.25	15.57	14.19
12.	PYPS-12	PSLRC 4 × PSLRC 12	26.80^ab^	5.20^ef^	44.80^k^	52.40^ij^	23.50	4.00	2.75	8.71
13.	PYPS-13	PSLRC 2 × PSLRC 21	19.80^efg^	7.90^ab^	64.50^c^	58.50^g^	6.45	−3.66	13.56	14.93
14.	PYPS-14	PSLRC 20 × PSLRC 21	20.54^ef^	6.24^bcdef^	60.34^d^	66.84^c^	5.12	−4.59	3.61	−3.61
15.	PYPS-15	PSLRC 2 × PSLRC 7	23.14^c^	7.24^abc^	72.14^b^	51.54^jk^	6.93	−5.24	8.25	15.98
16.	PYPS-16	PSLRC 20 × PSLRC 7	18.41^gh^	6.01^bcdef^	55.81^f^	59.81^f^	−4.16	11.09	−1.06	16.57
17.	PYPS-17	PSLRC 2 × PSLRC 8	17.22^h^	7.32^abcd^	73.22^a^	46.32^o^	2.38	1.39	9.58	12.37
18.	PYPS-18	PSLRC 3 × PSLRC 8	18.55^gh^	7.55^abc^	60.85^d^	52.45^ij^	−3.64	7.09	10.14	14.90
19.	PYPS-19	PSLRC 4 × PSLRC 8	21.28^de^	7.08^abcde^	56.18^f^	50.78^kl^	−7.80	12.74	8.92	9.96
	CD		0.87	0.84	0.84	0.84				
	CV		2.56	7.76	0.86	0.90				
	General Mean:		20.48	6.55	58.69	56.33				

Means followed by a common letter are not significantly different at 5% level.

#### Total phenolics, tannins and antioxidant activity

The total phenolic content showed significant (*p* < 0.05) variation ranging from 44.07 mg/g GAE (PYPS 3) to 85.65 mg/g GAE (PYPS 18 (Table 4). Significant (*p* < 0.05) variations were also noted in the tannins and anti-oxidant activity of the 19 sorghum genotypes. PYPS 15 (38.34 mg/g) and PYPS 9 (37.82 mg/g) showed significantly (*p* < 0.05) higher tannin content and PYPS 12 (22.10 mg/g) and PYPS 5 (17.78 mg/g) showed significantly (*p* < 0.05) lower tannins (Table 4). The anti-oxidant activity also showed significant variation (*p* < 0.05) among the genotypes ranging from 43.55% (PYPS 3) to 89.78% (PYPS 19).

**Table 4:** Improvement of phenolics, tannins and anti-oxidant activity in sorghum developed by single seed descent method during 2017–2022.

S. No.	Genotype	Pedigree	Developed during 2022			% Improvement due to selection		
			Phenolics (mg/g GAE)	Tannins (mg/g)	Anti-oxidant activity (%)	Phenolics	Tannins	Anti-oxidant activity
1.	PYPS-1	PSLRC 2 × PSLRC 3	66.43^h^	30.82^d^	79.32^c^	9.21	17.10	7.45
2.	PYPS-2	PSLRC 2 × PSLRC 4	75.27^c^	36.31^b^	83.41^b^	7.12	14.15	−1.42
3.	PYPS-3	PSLRC 3 × PSLRC 4	44.07^h^	28.15^f^	43.55^p^	9.44	−3.76	5.58
4.	PYPS-4	PSLRC 2 × PSLRC 6	69.35^f^	31.25^d^	70.65^gh^	12.31	3.65	−1.53
5.	PYPS-5	PSLRC 21 × PSLRC 7	48.40^l^	17.78^j^	52.78^m^	13.88	8.55	4.97
6.	PYPS-6	PSLRC 3 × PSLRC 6	52.50^k^	23.50^h^	56.10^k^	5.00	8.29	9.57
7.	PYPS-7	PSLRC 3 × PSLRC 7	58.34^i^	29.54^e^	48.84^n^	13.86	10.47	11.92
8.	PYPS-8	PSLRC 8 × PSLRC 9	66.16^h^	33.46^c^	76.16^d^	0.61	5.35	3.82
9.	PYPS-9	PSLRC 8 × PSLRC 10	68.12^g^	37.82^a^	71.52^fg^	11.09	19.61	4.68
10.	PYPS-10	PSLRC 9 × PSLRC 10	70.52^e^	31.22^d^	74.82^e^	18.48	13.86	15.43
11.	PYPS-11	PSLRC 4 × PSLRC 6	46.22^m^	23.81^h^	53.11^m^	6.94	13.33	2.11
12.	PYPS-12	PSLRC 4 × PSLRC 12	58.31^i^	22.10^i^	46.60^o^	14.99	6.25	7.87
13.	PYPS-13	PSLRC 2 × PSLRC 21	77.80^b^	36.20^b^	70.30^h^	10.67	1.97	−6.64
14.	PYPS-14	PSLRC 20 × PSLRC 21	72.64^d^	30.94^d^	72.34^f^	−0.68	15.28	1.26
15.	PYPS-15	PSLRC 2 × PSLRC 7	73.04^d^	38.34^a^	70.84^gh^	−3.44	21.18	6.78
16.	PYPS-16	PSLRC 20 × PSLRC 7	57.41^j^	23.61^h^	67.21^j^	11.24	−4.84	8.74
17.	PYPS-17	PSLRC 2 × PSLRC 8	66.32^h^	31.72^d^	68.82^j^	7.28	10.83	−0.72
18.	PYPS-18	PSLRC 3 × PSLRC 8	85.65^a^	25.65^g^	54.25^i^	10.87	6.65	4.63
19.	PYPS-19	PSLRC 4 × PSLRC 8	48.30^l^	26.28^g^	89.78^a^	13.38	20.66	10.46
	CD		0.84	0.87	0.87			
	CV		0.80	1.79	0.80			
	General Mean:		63.41	29.35	65.81			

Means followed by a common letter are not significantly different at 5% level.

### Improvement in nutrient and quality parameters due to SSD

The 19 sorghum genotypes that were developed by crossing 11 sorghum landraces were continuously selected from 2017 until 2022. The changes in nine nutritional and quality parameters in sorghum grains were determined every year and the overall improvement by the year 2022 is summarized here.

#### Crude protein and IVPD

All the 19 sorghum genotypes showed improvement with a positive increase in the crude protein content (Table 2). Sorghum genotypes PYPS 10 (32.35%), PYPS 6 (31.66%) and PYPS 3 (27.01%) showed highest improvement and genotypes PYPS 1 (7.24%) and PYPS 12 (1.84%) showed lowest improvement (Table 2). In the IVPD, all the genotypes showed an increase compared to the values during 2017 at the beginning of the selection except for the genotype PYPS 2, which had a reduced IVPD by 5.75% (Table 2).

#### Total and available Fe and Zn

Out of the 19 sorghum genotypes, 13 genotypes had an improvement in their total Fe content with PYPS 12 (23.50%), PYPS 3 (16.43%) and PYPS 8 (14.50%) showing the highest improvement during 2022 compared to 2017. Genotypes PYPS 17 (−5.68%), PYPS 10 (−5.78%) and PYPS 19 (−7.80%) had reduced Fe content during 2022 compared to 2017 (Table 2). The available Fe showed improvement in all the sorghum genotypes except for PYPS 16 which had a reduced available Fe content by 1.06% (Table 2). Genotypes PYPS 3 (26.79%), PYPS 5 (18.02%) and PYPS 11 (15.57%) had highest improvement in their available Fe contents (Table 2). Eight genotypes had a reduction in their available Zn with PYPS 6 (−7.81%) showing the highest reduction followed by the genotypes PYPS 9 (−5.54%) and PYPS 15 (−5.24%) (Table 2). On the other hand, only one genotype PYPS 14 (−3.61%) showed a reduction in the total available Zn during 2022 compared to 2017. Highest improvement was observed in PYPS 7 (17.30%), PYPS 16 (16.57%) and PYPS 15 (15.98%; Table 2).

#### Total phenolics, tannins and antioxidant activity

The genotypes also varied differently in their total phenolics, tannins and anti-oxidant activity (Table 4). While two genotypes PYPS 14 and PYPS 15 showed a reduction in their total phenolics by 0.68 and 3.44% respectively, genotypes PYPS 3 and PYPS 16 showed reduced tannin levels by 3.76 and 4.84%, respectively, (Table 4). Four genotypes PYPS 2, PYPS 4, PYPS 13 and PYPS 17 had the antioxidant activity reduced by 1.42, 1.53, 6.64 and 0.72%, respectively. Highest improvement in total phenolics was observed in PYPS 10 (18.48%) and PYPS 12 (14.99%) while the highest improvement in tannins was observed in PYPS 15 (21.18%) and PYPS 19 (20.66%). The anti-oxidant activity showed high improvement in PYPS 10 (15.43%) and PYPS 7 (11.92%).

### PCA biplot

**Figure 1:**
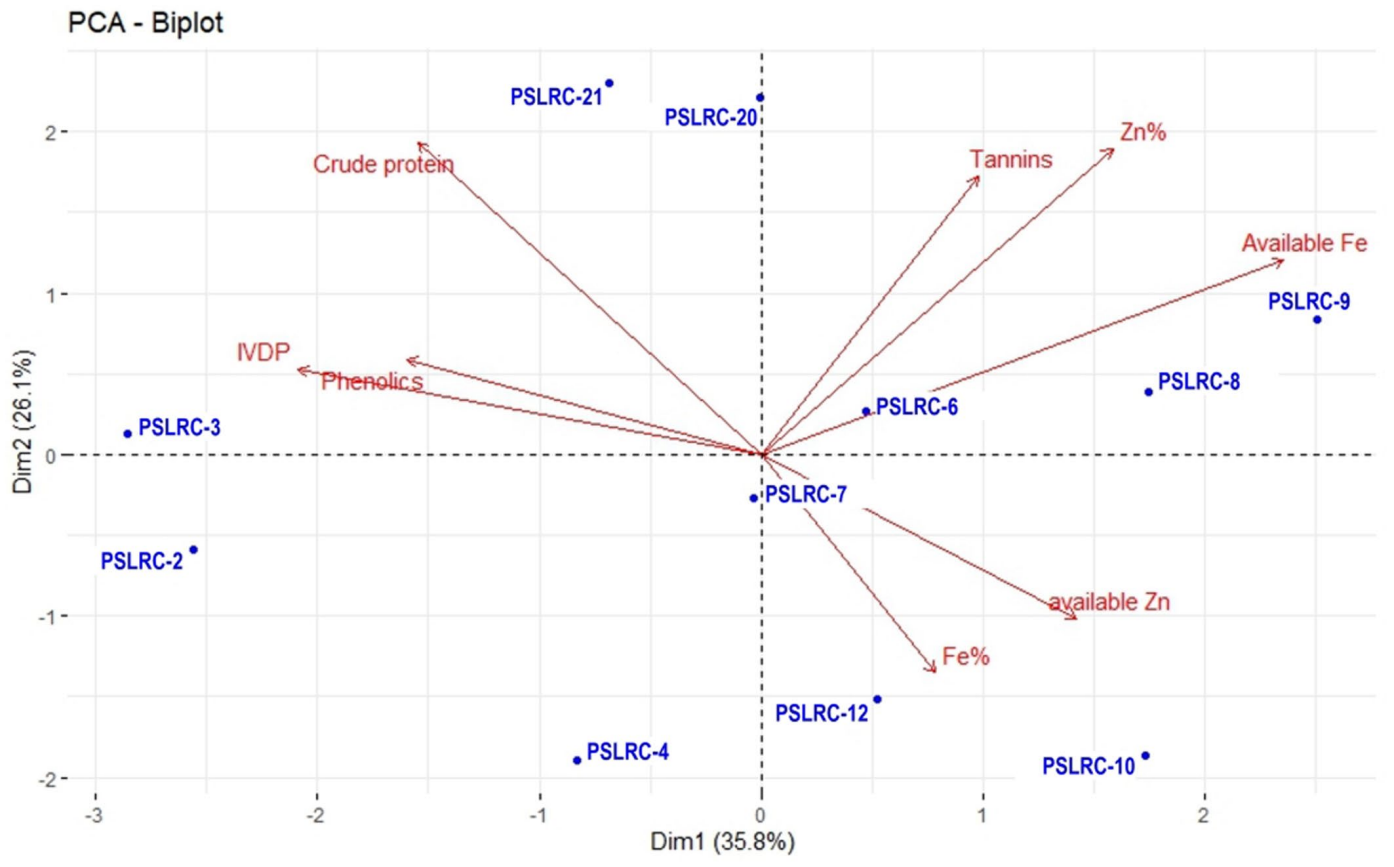
PCA biplot of nutritional and quality characters of 11 sorghum landraces used in the study.

**Figure 2:**
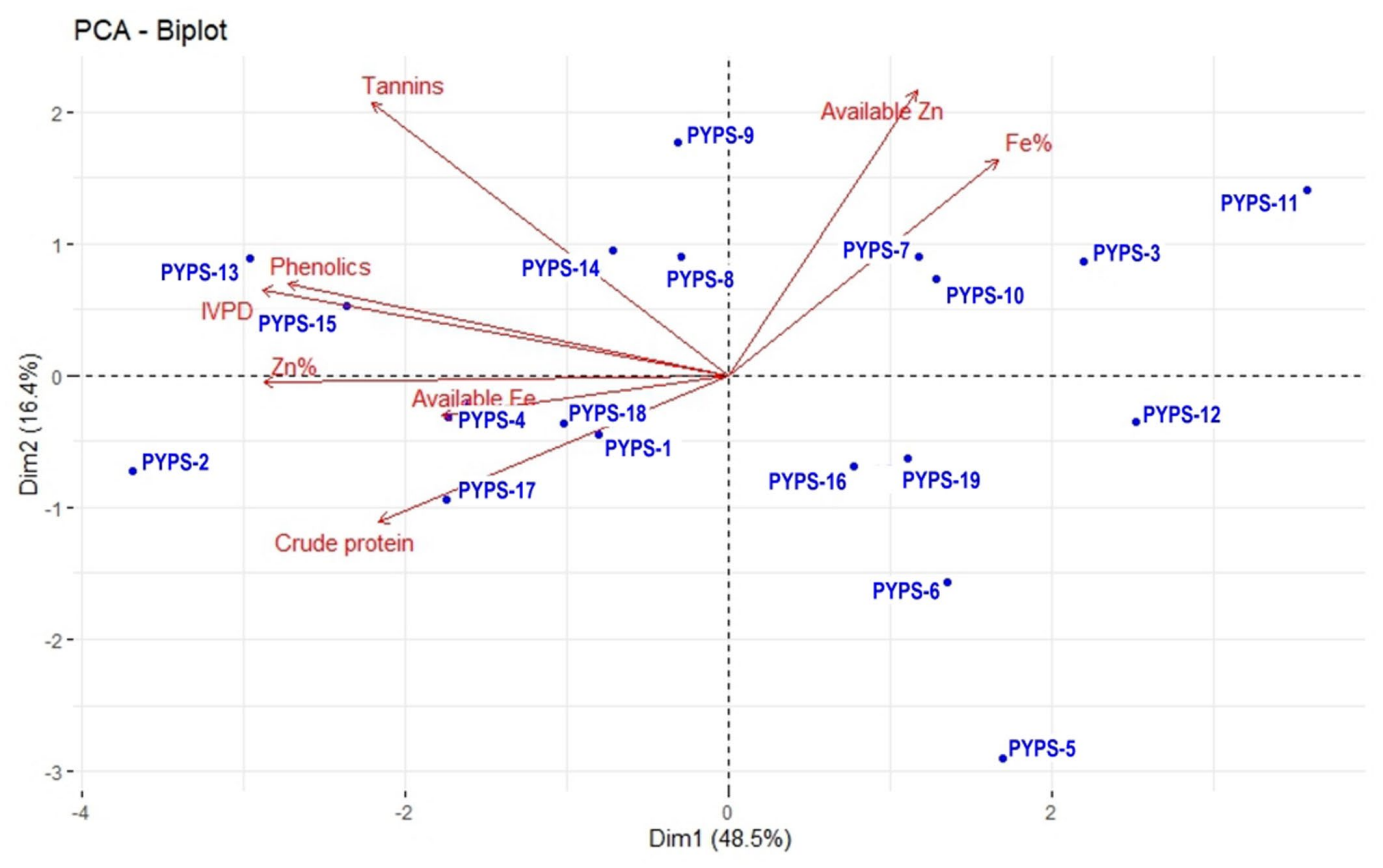
PCA biplot of nutritional and quality characters of 19 sorghum genotypes during 2022 evolved using 11 sorghum landraces.

Two PCA biplots were generated with all the nutritional parameters in sorghum landraces (Figure 1) and sorghum genotypes developed from the landraces during 2022 (Figure 2). The first two dimensions explained 61.9% of the total variability in the nutritional profiles of the 11 sorghum landraces (Figure 1). The PC1 was mainly represented by available Fe and total Zn, while PC2 was mostly characterized by tannins and crude protein. Tannins, total Zn and available Fe showed similar trends and so did crude protein, IVDP, and phenolics. However, crude protein showed a negative correlation with the available Zn and total Fe. Except for PSLRC-6 and PSLRC-7, all the other 9 landraces were largely dispersed and away from origin inferring high variability and their importance in selection. Landraces differed in their performance in the nutritional characteristics. For example, landraces PSLRC 6, 8 and 7 showed better performance for available Fe and PSLRC 7 and 9 showed better performance for total Fe; For IVPD and phenolics, better performance was observed for landrace PSLRC 3; landrace PSLRC 21 showed better performance for crude protein (Figure 1).

In the PCA biplot for the nutritional characters of the 19 sorghum genotypes developed from the landraces, the first two dimensions explained 64.9% of the total nutritional variability (Figure 2). The PC1 was mainly represented by total Fe and available Zn and PC2 was mostly characterized by tannins and phenolics. Available Zn and total Fe showed a similar trend and so did the tannins, phenolics and IVPD. Similarly, total Zn, available Fe and crude protein also showed a similar trend. However, crude protein showed a negative correlation with the available Zn and total Fe. Sorghum genotypes PYPS 2, PYPS 3, PYPS 5, PYPS 6, PYPS 11, PYPS 12, PYPS 13 and PYPS 15 were largely dispersed and away from the origin indicating their high variability in the evaluated nutritional parameters (Figure 2). Sorghum genotype PYPS 7 performed better for the Fe% and PYPS 13 showed better performance for phenolics. For IVPD and available Fe, genotypes PYPS 15 and PYPS 4 showed better performance. Two genotypes PYPS 1 and PYPS 17 performed better for crude protein (Figure 2).

### Correlations among the nutritional characters

**Figure 3:**
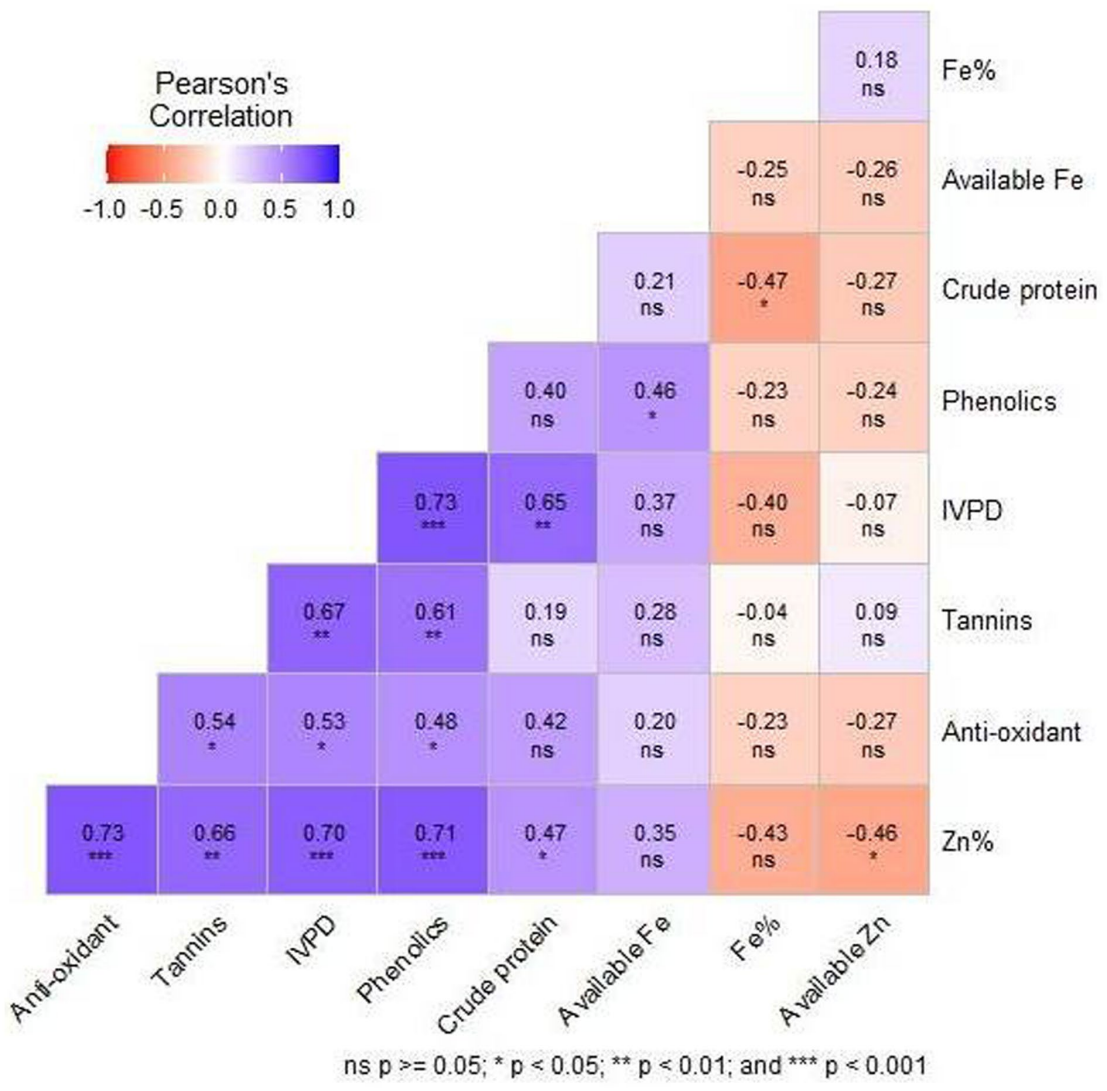
Pearson’s correlation of the 9 nutritional and quality characters of the 19 sorghum genotypes during 2022 evolved using 11 sorghum landraces.

Correlation coefficients between the nutritional parameters of 19 sorghum genotypes showed positive, negative and weak correlations among all the nutritional characters. Highly significant (*p* < 0.001) positive correlation was observed for total Zn with antioxidant activity (*R* = 0.73), phenolics (*R* = 0.71) and IVPD (*R* = 0.70). Phenolics showed a highly significant (*p* < 0.001) positive correlation with IVPD (*R* = 0.73). Significant (*p* < 0.01) positive correlation was also observed between IVPD and crude protein (*R* = 0.65), tannins and phenolics (*R* = 0.61), tannins and IVPD (*R* = 0.67), total Zn and tannins (*R* = 0.66%). Significant (*p* < 0.05) negative correlation was observed between crude protein and total Fe (*R* = −0.47), total Zn and available Zn (*R* = −0.46) (Figure 3).

## Discussion

This study is a part of the long-term sorghum breeding program to develop high-yielding, dual purpose, drought-tolerant, grain mold tolerant varieties with high nutritional and quality characters for nutritional and health security of the farmers in the dryland regions of Telangana, India. The study began with a set of 36 landraces collected from the interior regions of southern and central India during 2008 followed by single-plant selection to identify 11 diverse agronomically-superior and grain mold-tolerant landraces (18). In this study, 19 genotypes were evolved using these 11 sorghum landraces. Wide variation for protein, IVPD, Fe, Zn, tannins, total phenolics and anti-oxidant activity in landraces and their genotypes indicated the existence of genetic variability. Two conventional breeding methods based on hybridization viz., pedigree method and SSD method were employed in this study. First, a pedigree breeding method was used to develop 19 genotypes using 11 landraces for high yield and grain mold tolerance followed by the SSD method for nutritional improvement. Pedigree method is widely practiced method employed in sorghum improvement and many present-day varieties such as CSV 15, CSV 17, and CSV 20 are the outcomes of this method (33). In other cereals like rice, though pedigree breeding is the most commonly used method (34), the SSD method (also referred to as rapid generation advance) is the most feasible method for accelerated breeding by about 2 years (35). In this study, using SSD method, the Fe and Zn contents showed an overall improvement due to continuous selection, which is more effective in the next generation of lines obtained from the SSD method as an increase in the homozygosity results in increased variability between the lines due to additive gene action (36).

Protein is one of the most important components determining the nutritional value of food. Angelo (37), Elbashir et al., (38), Ahmed et al., (39) and Abdelhalim et al., (40) reported the crude protein of sorghum to be between 8 and 12.5% depending on the landrace or variety The reported crude protein was within the range of the findings of the current study with two genotypes PYPS 2 and PYPS 13 showing high crude protein contents of 13.21 and 12.80%, respectively. These values were higher than the requirements for daily maintenance (0.7–0.8%) in humans (41). Kulamarva et al. (42) reported that sorghum protein content may be affected by both genetics and environmental factors, which could be the reason for variations among the different landraces and genotypes. Compared to 2017, all the genotypes showed improved protein contents in 2022, some to the magnitude of 32%. Genetic variation in protein digestibility was also reported in sorghum (43) similar to the present study. These might be due to differences in the disulfide bonds between the cysteine residues of the seed proteins and the differing nature of the protein matrix in the endosperm. Resistance of storage proteins (kafirins) to proteolytic digestion also reduces starch digestibility because undigested kafirins prevent access of amylolytic enzymes to starch granules (9–11). Development of grain sorghum lines with improved digestibility of kafirins similar to that of other cereals will enhance the nutritional value of sorghum grain. Recently, Elkonin et al. (44) obtained grain sorghum mutants with improved kafirin digestibility using genome editing to enhance the nutritional value of sorghum grain.

In this study, we have reported landraces with high Zn and Fe contents ranging from 12.01 to 16.46 mg/100 and 4.72 to 6.84 mg/100 g, respectively. In line with the present study, earlier reports also indicated a wide range of values for Fe (1.10–9.54 and 3.00–11.30 mg/100 g) and Zn (1.10–5.02 and 1.12–7.58 mg/100 g) in sorghum landraces (45, 46). Comparatively, the 19 genotypes from the landraces recorded higher values ranging between 14.21 and 28.41 mg/100 g for Fe and 4.81–8.16 mg/100 g for Zn content. High heritabilities for Fe and Zn in landraces might have contributed to improved values in the genotypes (45).In crops like sorghum, in addition to nutritional content, it is also important to consider consumer’s accessibility to the nutrients where naturally occurring anti-nutritive factors vary and reduce the nutritional value compared to wheat or maize (47). These include phytic acid, phenolic compounds, enzyme inhibitors etc. which reduce the digestibility of the grain (47). For instance, phytic acid chelates positively charged minerals like Fe and Zn, forming insoluble complexes and causing decreased mineral absorption, in addition to forming complexes with protein, thus slowing the digestion rate and hampering their bio-availability (48). Several low phytic acid-containing landraces such as Malkhed-1 and Nalwar-2 and a variety Phule Maulee were reported (49). Due to these anti-nutrient interactions and the limited bio-availability of sorghum compared with other grains (50), harnessing the potential of nutrient-rich landraces and genotypes such as PSLRC-2, PSLRC-3, PSLRC-9, PYPS 2 and PYPS 13 by reducing the anti-nutritive factors should be an important goal especially in the arid and semi-arid regions of Asia and Sub-Saharan Africa, where malnutrition and nutritional deficiencies are prevalent in women and children.

Correlations between traits are important for the success of selections in a breeding program for nutrient improvement without compromising the yield potential. In the present study, similar to the findings of Motlhaodi et al. (51), Zn and crude protein were significantly correlated, indicating common genomic regions or genes or biochemical pathways involved in the expression of these traits (52). Positive correlations between these traits were also reported in wheat (53), pearl millet (54), rice (55) and in sorghum (56) indicating the possibility of combining Zn and crude protein in a single agronomic background and for simultaneous selection of these two traits. Genetic mapping in different wheat populations confirmed co-localization of quantitative trait loci (QTL) conferring high protein, high Zn and high Fe (57). Similarly, co-localization of QTLs for Zn and Fe concentrations was reported in rice (58). On the other hand, Zn and available Zn were negatively correlated and this might be because Zn is concentrated in the aleurone and the scutellum of the embryo and are co-located with phosphate in the form of salts of phytic acid, which have low solubility and hence low bio-availability, thus clearly demonstrating that distribution and form of minerals have implications for grain processing and human health (59). In addition to an understanding of genetic control, breeding for the enrichment of these micronutrients also requires knowledge of the phenotypic association, environments and interactions. Phuke et al. (52) reported the significant effects of the environment on the amounts of micronutrients in sorghum. Alteration of the environment affects the physiology of the sorghum thereby affecting the uptake and accumulation of minerals in the plant (60–65). The present study was conducted at R.A.R.S., Palem, which is the heartland for cultivation of dryland sorghum in Telangana state in India. Because there is a great variation in the soil micronutrient status in dryland Telangana (66), the genotype × environment interaction for sorghum grain nutrients might be large requiring tools like GGE biplot for characterizing and grouping the environments (67).

Sorghum has a diverse range of phenolic compounds not found in other cereal grains and some compounds like 3-deoxyanthocyanins, are not known to exist in any other edible plants (68). Sorghum phenolic compounds have been reported to possess important and unique bioactive properties relevant to cancer prevention, cardiovascular health and reduced chronic inflammation and oxidative stress etc, (69, 70). In the present study, a wide range of values for phenolics was observed in landraces and their genotypes with varied improvements due to selections during 2022 compared to 2017. The composition of the specific phenolics in sorghum in highly variety-dependent with well-characterized genes controlling their synthesis (71, 72) making it possible for selection and genetic improvement of sorghum for targeted applications in food for health and quality. A significant positive correlation between the anti-oxidant activity and phenolics is found in the present study. This might be because of the presence of unique polyphenols like 3-deoxyanthocyanins which are strong inducers of detoxifying enzymes such as phase II enzymes with consequences anti-oxidant capacity (69).

In this study, we utilized the genetic diversity of sorghum landraces and developed new genotypes with improved nutritional content. Similar studies in other cereals like wheat and rice also demonstrated the role of landraces in breeding new cultivars with high nutritional value. For example, in wheat, the landraces of Portugal and Spain had genetic variability in high molecular glutenin subunits (73) and in rice, 22 novel genomic loci were identified for Zn, Fe and oleic acid etc. (74). The landraces used in the present study showed wide variation in crude protein, IVPD, total and available Fe and Zn, total phenolics, tannins and anti-oxidant activity suggesting that they might be a reservoir of important and unique genes, thus corroborating their role in sorghum improvement program. Compared to the landraces, the genotypes showed wide variation in their nutritional values with varying magnitudes of improvement during 2017–2022 suggesting differences in their expression. Identifying the genes influencing these traits might help sorghum breeders to further improve the grain quality. This is important, especially for those characters with closely linked molecular markers for developing new varieties with high yield and nutritional value (16).

Overall, an attempt was made to develop biofortified sorghum lines by utilizing the genetic variability of sorghum landraces and characterizing the nutritional profiles. Genotypic stability is crucial not only for grain yield but also for nutritional traits and the best genotype needs to combine good yield and stable performance across a range of production environments. High protein containing PYPS 2 and PYPS 13 were previously identified as the most stable genotypes with high grain (3,698 kg/ha and 3,514 kg/ha respectively) and high fodder (20,585 kg/ha and 18,122 kg/ha respectively) yields during multi-environment testing across the drought-prone regions of Telangana (18). On the other hand, the study also identified high Fe-containing genotypes PYPS 8 and PYPS 11 as the most unstable genotypes for yield. This validates the importance of investigating possible G × E interaction by multi-environment testing to identify high-yielding, nutrient-rich stable sorghum genotypes suitable for rainfed cultivation in Asia and Africa.

Sorghum is a cheap source of energy and micronutrients in India and sub-Saharan Africa, where Fe and Zn deficiencies are reported in over three billion people worldwide (75). Biofortification (76) is a sustainable and cost-effective approach for improving the nutritional quality of food and for catering to more than half the dietary micronutrients of the low-income group of rural India, where economic access to nutrient-rich food is limited (77, 78). The study identified high protein, Fe and Zn-containing landraces such as PSLRC-6, PSLRC-9, PSLRC-10 and PSLRC-20 and utilized them to develop high protein and micronutrients containing genotypes such as PYPS 2, PYPS 11 and PYPS 13, thus corroborating the use of landraces a promising source for biofortification. Further, significant variations in the nutritional parameters, and major improvements, were also demonstrated using the SSD breeding method. These variations could be genetically controlled, environmentally influenced or both with possible metabolic trade-offs, where changes in one trait might have consequences on the other traits. For example, pigmented testa in sorghum contributes to high tannins conferring resistance to grain mold disease while also simultaneously hindering IVPD in both animals and humans (79, 80). Hence, it is important to understand the underlying causes of these variations in grain nutritional profile to guide future breeding to optimize the improvement of specific nutritional traits in sorghum while minimizing the unintended effects on other parameters.

## Conclusion

Sorghum is one of large contributions to the recommended dietary allowances of the population living in the arid and semi-arid regions of Asia and Sub-Saharan Africa. Nutrient profiling of sorghum landraces has revealed several of them with good sources of nutrients but not likely on the modern varieties and hybrids. Significantly higher variability among landraces for nutritional and yield contributes to improvement through conventional plant breeding prospecting biofortification in sorghum. High-protein genotypes PYPS 2 and PYPS 13 evolved from high-protein containing landraces PSLRC 2 and PSLRC 21 reinforced the importance of selection of landraces to develop biofortified sorghum. Changes in crude protein (PYPS 3, PYPS 6, PYPS 10), Fe (PYPS 3, PYPS 8, PYPS 12) and Zn (PYPS 7, PYPS 15, PYPS 16) during SSD contents clearly demonstrated the importance of selection in grain sorghum nutritional improvement. Identified genotypes with better nutritional profiles combined with less anti-nutritional content would be the best parents in early hybridization and pipelines strategy for AICRP-sorghum breeding programs in India. Future interventions should focus on utilizing superior landraces to develop competitive biofortified sorghum, especially for the resource-poor farmers of India and elsewhere who subsist on sorghum for productivity and nutritional security.

## Data availability statement

The original contributions presented in the study are included in the article/Supplementary material, further inquiries can be directed to the corresponding author.

## Author contributions

NM, RV, MS, and GN designed and carried out the experiments. NM, RV, MG, and VK analyzed the data. RV, MS, and KK conducted the field experiments with logistical support from GN. NM, RV, and MG wrote the draft manuscript. All authors made contributions toward the compilation of the draft and approved the final manuscript.
