# Supplementary material for: Exploiting Indian landraces to develop biofortified grain sorghum with high protein and minerals
Source: Front Nutr. 2023 Oct 9;10:1228422. doi: 10.3389/fnut.2023.1228422 (PMC10591322; doi:10.3389/fnut.2023.1228422)
Supplement: Supplementary file 1 [file Data_Sheet_1.docx]

**SUPPLEMENTARY TABLE S1:** Details of 36 sorghum landraces collected from various parts of southern and central India during 2008 including the 11 landraces used in the study

| **S. No.** | **Local name** | **State** | **Location/District** | **Accession Identifier given by ICAR-NBPGR** | **Days to 50% Flowering** | **Panicle compactness** | **Grain Color** | **Accession Identifier given under the project** |
| --- | --- | --- | --- | --- | --- | --- | --- | --- |
| 1 | Chitta patchajonna | Andhra Pradesh | Kurnool | IS-5065 | 66 | Semi compact | Yellow | PSLRC 7 |
| 2 | Gundu patch jonna | Andhra Pradesh | Nandyal | IS-1138 | 71 | Semi compact | Yellow | PSLRC 1 |
| 3 | Gundu patchajonna | Andhra Pradesh | Kodumuru | IS-21979 | 68 | Semi compact | Yellow | PSLRC 2 |
| 4 | Jowarilangpur | Telangana | Adilabad | IS-5244 | 62 | Semi compact | Yellow | PSLRC 14 |
| 5 | Pedda patchajonna | Andhra Pradesh | Nellore | IS-17789 | 60 | Semi compact | Yellow | PSLRC 4 |
| 6 | Pyrupatchajonna | Andhra Pradesh | Prakasam | IS-17796 | 70 | Compact | Yellow | PSLRC 6 |
| 7 | Rangampetjonna | Telangana | Medak | IS-5276 | 64 | Semi compact | Yellow | PSLRC 13 |
| 8 | Tekedarijonna | Telangana | Adilabad | IS-33623 | 66 | Compact | Yellow | PSLRC 30 |
| 9 | Yellow cholam | Andhra Pradesh | Kurnool | IS-5068 | 60 | Compact | Yellow | PSLRC 12 |
| 10 | Yellow jowar | Telangana | Khammam | IS-17660 | 58 | Semi compact | Yellow | PSLRC 28 |
| 11 | Argadijola | Karnataka | Bidar | IS-5583 | 58 | Compact | Yellow | PSLRC 32 |
| 12 | Bhagwathijola | Karnataka | Bijapur | IS-5639 | 65 | Compact | Yellow | PSLRC 16 |
| 13 | Bhangar kaddi | Karnataka | Raichur | IS-5552 | 62 | Compact | Yellow | PSLRC 34 |
| 14 | Patch jonna | Karnataka | Bidar | IS-22185 | 68 | Semi compact | Yellow | PSLRC 33 |
| 15 | Yellow chickni Khajijot | Maharashtra | E.Khandesh | IS-4922 | 65 | Semi compact | Yellow | PSLRC 18 |
| 16 | Yellow wani | Maharashtra | Akola | IS-17994 | 60 | Semi compact | Yellow | PSLRC 26 |
| 17 | Peeli Maharashtra | Maharashtra |  | IS-24366 | 65 | Compact | Yellow | PSLRC 19 |
| 18 | Palam jonna | Telangana | Adilabad | IS-33658 | 64 | Compact | Yellow | PSLRC 5 |
| 19 | Peelikalgondi | Maharashtra | Nanded | IS-4606 | 66 | Semi compact | Yellow | PSLRC 35 |
| 20 | Peelitarangri | Maharashtra | Parbhani | IS-4568 | 60 | Semi compact | Yellow | PSLRC 36 |
| 21 | Gingri jowar | Telangana | Adilabad | IS-33665 | 62 | Semi compact | Brown | PSLRC 8 |
| 22 | Pyruyerrajonna | Andhra Pradesh | West Godavari | IS-5152 | 68 | Compact | Brown | PSLRC 9 |
| 23 | Red jowar | Telangana | Khammam | IS-17687 | 60 | Compact | Brown | PSLRC 10 |
| 24 | Yerra jonna | Andhra Pradesh | Kadapa | IS-17800 | 60 | Compact | Brown | PSLRC 15 |
| 25 | Yerra mallejonna | Andhra Pradesh | Adilabad | IS-33636 | 70 | Semi compact | Brown | PSLRC 11 |
| 26 | Pandhari jowar | Maharashtra | Sholapur | IS-33761 | 65 | Compact | Brown | PSLRC 17 |
| 27 | Lal gunja | Maharashtra | Buldana | IS-4977 | 72 | Compact | Brown | PSLRC 23 |
| 28 | Lal jowar | Maharashtra | Solapur | IS-33760 | 70 | Compact | Brown | PSLRC 24 |
| 29 | Lal shallu | Maharashtra | Aurangabad | IS-40738 | 77 | Compact | Brown | PSLRC 25 |
| 30 | Lalbhindikolumbi | Maharashtra | Nanded | IS-4599 | 72 | Compact | Brown | PSLRC 27 |
| 31 | Lalgunjachirode | Maharashtra | Amaravati | IS-5008 | 72 | Compact | Brown | PSLRC 31 |
| 32 | Sai jonna | Telangana | Adilabad | IS-33686 | 65 | Semi compact | White | PSLRC 3 |
| 33 | Tandur Local | Telangana | Ranga Reddy | IS-36451 | 60 | Semi compact | White | PSLRC 29 |
| 34 | Kaki jonna | Telangana |  | IS-22197 | 66 | Semi compact | White black glume | PSLRC 22 |
| 35 | Nila jonna | Telangana | Karimnagar | IS-5238 | 66 | Loose | White black glume | PSLRC 21 |
| 36 | Pandri jowar | Maharashtra | Parbhani | IS-40784 | 68 | Semi compact | White black glume | PSLRC 20 |

**SUPPLEMENTARY TABLE S2:** Crude protein (%) in the grains of sorghum genotypes evolved from landraces during 2017- 2022

| **S. No.** | **Genotype** | **Pedigree** | **2017** | **2018** | **2019** | **2020** | **2021** | **2022** | **Mean** |
| --- | --- | --- | --- | --- | --- | --- | --- | --- | --- |
| 1. | PYPS-1 | PSLRC 2 x PSLRC 3 | 11.05 | 11.81 | 10.66 | 11.59 | 11.48 | 11.85 | 11.41 |
| 2. | PYPS-2 | PSLRC 2 x PSLRC 4 | 11.96 | 12.32 | 12.10 | 11.81 | 12.58 | 13.21 | 12.33 |
| 3. | PYPS-3 | PSLRC 3 x PSLRC 4 | 8.22 | 8.18 | 8.32 | 8.24 | 9.79 | 10.44 | 8.87 |
| 4. | PYPS-4 | PSLRC 2 x PSLRC 6 | 9.05 | 9.21 | 9.31 | 9.59 | 10.25 | 10.26 | 9.61 |
| 5. | PYPS-5 | PSLRC 21 x PSLRC 7 | 10.26 | 10.32 | 10.54 | 10.88 | 11.28 | 11.73 | 10.84 |
| 6. | PYPS-6 | PSLRC 3 x PSLRC 6 | 7.96 | 8.47 | 8.82 | 9.56 | 10.16 | 10.48 | 9.24 |
| 7. | PYPS-7 | PSLRC 3 x PSLRC 7 | 8.09 | 8.42 | 8.68 | 8.95 | 9.46 | 9.67 | 8.88 |
| 8. | PYPS-8 | PSLRC 8 x PSLRC 9 | 7.48 | 8.20 | 8.42 | 8.91 | 9.17 | 8.94 | 8.52 |
| 9. | PYPS-9 | PSLRC 8 x PSLRC 10 | 7.67 | 7.84 | 7.98 | 8.42 | 8.95 | 9.59 | 8.41 |
| 10. | PYPS-10 | PSLRC 9 x PSLRC 10 | 7.07 | 7.77 | 8.63 | 9.20 | 9.27 | 9.35 | 8.55 |
| 11. | PYPS-11 | PSLRC 4 x PSLRC 6 | 8.32 | 8.46 | 8.57 | 8.52 | 8.64 | 8.79 | 8.55 |
| 12. | PYPS-12 | PSLRC 4 x PSLRC 12 | 9.25 | 9.16 | 9.30 | 9.38 | 9.56 | 9.42 | 9.35 |
| 13. | PYPS-13 | PSLRC 2 x PSLRC 21 | 11.20 | 11.35 | 11.56 | 11.78 | 12.60 | 12.80 | 11.88 |
| 14. | PYPS-14 | PSLRC 20 x PSLRC 21 | 10.12 | 10.78 | 10.92 | 11.36 | 11.85 | 11.67 | 11.19 |
| 15. | PYPS-15 | PSLRC 2 x PSLRC 7 | 10.12 | 10.84 | 11.14 | 11.49 | 11.74 | 11.76 | 11.16 |
| 16. | PYPS-16 | PSLRC 20 x PSLRC 7 | 9.37 | 9.88 | 9.87 | 10.43 | 10.66 | 10.60 | 10.14 |
| 17. | PYPS-17 | PSLRC 2 x PSLRC 8 | 10.24 | 10.58 | 10.66 | 10.91 | 11.24 | 11.39 | 10.84 |
| 18. | PYPS-18 | PSLRC 3 x PSLRC 8 | 8.00 | 8.42 | 8.55 | 8.62 | 9.27 | 9.52 | 8.73 |
| 19. | PYPS-19 | PSLRC 4 x PSLRC 8 | 8.25 | 8.81 | 8.88 | 9.22 | 9.48 | 9.68 | 9.05 |
|  | CD |  | 0.97 | 0.97 | 0.97 | 0.97 | 0.97 | 0.97 | 0.97 |
|  | CV |  | 6.42 | 6.18 | 6.11 | 5.91 | 5.66 | 5.55 | 5.95 |
|  | General Mean: |  | 9.16 | 9.51 | 9.63 | 9.94 | 10.39 | 10.59 |  |

**SUPPLEMENTARY TABLE S3:** In vitro protein digestibility (%) in the grains of sorghum genotypes evolved from landraces during 2017- 2022

| **S. No.** | **Genotype** | **Pedigree** | **2017** | **2018** | **2019** | **2020** | **2021** | **2022** | **Mean** |
| --- | --- | --- | --- | --- | --- | --- | --- | --- | --- |
| 1. | PYPS-1 | PSLRC 2 x PSLRC 3 | 13.84 | 13.65 | 14.83 | 15.64 | 15.86 | 16.59 | 15.07 |
| 2. | PYPS-2 | PSLRC 2 x PSLRC 4 | 19.82 | 17.61 | 18.40 | 18.31 | 18.62 | 18.68 | 18.57 |
| 3. | PYPS-3 | PSLRC 3 x PSLRC 4 | 12.14 | 14.56 | 14.14 | 13.61 | 13.35 | 13.72 | 13.59 |
| 4. | PYPS-4 | PSLRC 2 x PSLRC 6 | 14.36 | 14.88 | 15.99 | 15.42 | 15.71 | 15.87 | 15.37 |
| 5. | PYPS-5 | PSLRC 21 x PSLRC 7 | 11.58 | 12.77 | 13.91 | 13.14 | 13.59 | 13.86 | 13.14 |
| 6. | PYPS-6 | PSLRC 3 x PSLRC 6 | 10.47 | 10.63 | 11.23 | 11.70 | 12.82 | 12.55 | 11.57 |
| 7. | PYPS-7 | PSLRC 3 x PSLRC 7 | 13.99 | 14.67 | 15.76 | 15.72 | 15.34 | 15.44 | 15.15 |
| 8. | PYPS-8 | PSLRC 8 x PSLRC 9 | 13.28 | 13.61 | 13.79 | 14.58 | 14.76 | 14.32 | 14.06 |
| 9. | PYPS-9 | PSLRC 8 x PSLRC 10 | 14.57 | 14.94 | 15.40 | 16.00 | 16.72 | 15.77 | 15.57 |
| 10. | PYPS-10 | PSLRC 9 x PSLRC 10 | 12.28 | 12.97 | 13.44 | 13.40 | 14.10 | 13.38 | 13.26 |
| 11. | PYPS-11 | PSLRC 4 x PSLRC 6 | 11.16 | 12.38 | 12.55 | 12.91 | 12.76 | 12.93 | 12.45 |
| 12. | PYPS-12 | PSLRC 4 x PSLRC 12 | 12.60 | 12.95 | 12.80 | 13.53 | 13.70 | 13.36 | 13.16 |
| 13. | PYPS-13 | PSLRC 2 x PSLRC 21 | 16.76 | 17.82 | 17.38 | 18.77 | 19.32 | 19.56 | 18.27 |
| 14. | PYPS-14 | PSLRC 20 x PSLRC 21 | 14.54 | 15.15 | 15.49 | 15.80 | 16.30 | 16.52 | 15.63 |
| 15. | PYPS-15 | PSLRC 2 x PSLRC 7 | 15.22 | 15.80 | 15.66 | 16.59 | 16.64 | 17.54 | 16.24 |
| 16. | PYPS-16 | PSLRC 20 x PSLRC 7 | 13.13 | 13.53 | 14.76 | 14.66 | 15.00 | 15.66 | 14.46 |
| 17. | PYPS-17 | PSLRC 2 x PSLRC 8 | 14.32 | 14.87 | 14.75 | 15.62 | 15.90 | 16.34 | 15.30 |
| 18. | PYPS-18 | PSLRC 3 x PSLRC 8 | 13.71 | 14.18 | 15.66 | 15.91 | 16.16 | 16.85 | 15.41 |
| 19. | PYPS-19 | PSLRC 4 x PSLRC 8 | 13.28 | 12.25 | 12.58 | 14.47 | 15.52 | 15.85 | 13.99 |
|  | CD |  | 0.97 | 0.97 | 0.97 | 0.97 | 0.97 | 0.84 | 0.97 |
|  | CV |  | 4.28 | 4.15 | 4.01 | 3.91 | 3.82 | 3.28 | 3.77 |
|  | General Mean: |  | 13.74 | 14.17 | 14.66 | 15.04 | 15.38 | 15.52 |  |

**SUPPLEMENTARY TABLE S4:** Fe content (mg/100g) in the grains of sorghum genotypes evolved from landraces during 2017- 2022

| **S. No.** | **Genotype** | **Pedigree** | **2017** | **2018** | **2019** | **2020** | **2021** | **2022** | **Mean** |
| --- | --- | --- | --- | --- | --- | --- | --- | --- | --- |
| 1. | PYPS-1 | PSLRC 2 x PSLRC 3 | 17.22 | 17.82 | 17.32 | 17.92 | 18.42 | 18.62 | 17.89 |
| 2. | PYPS-2 | PSLRC 2 x PSLRC 4 | 12.81 | 12.61 | 13.01 | 13.51 | 13.31 | 14.21 | 13.24 |
| 3. | PYPS-3 | PSLRC 3 x PSLRC 4 | 17.65 | 17.95 | 17.75 | 16.85 | 18.75 | 20.55 | 18.25 |
| 4. | PYPS-4 | PSLRC 2 x PSLRC 6 | 13.65 | 12.85 | 13.25 | 13.95 | 14.55 | 15.35 | 13.93 |
| 5. | PYPS-5 | PSLRC 21 x PSLRC 7 | 14.78 | 15.88 | 15.18 | 16.18 | 14.98 | 15.48 | 15.41 |
| 6. | PYPS-6 | PSLRC 3 x PSLRC 6 | 19.70 | 18.10 | 19.40 | 18.40 | 18.20 | 19.20 | 18.83 |
| 7. | PYPS-7 | PSLRC 3 x PSLRC 7 | 24.64 | 23.94 | 25.44 | 23.94 | 22.64 | 23.24 | 23.97 |
| 8. | PYPS-8 | PSLRC 8 x PSLRC 9 | 22.76 | 24.56 | 25.76 | 26.76 | 23.86 | 26.06 | 24.96 |
| 9. | PYPS-9 | PSLRC 8 x PSLRC 10 | 17.62 | 18.92 | 17.22 | 18.52 | 18.82 | 19.52 | 18.44 |
| 10. | PYPS-10 | PSLRC 9 x PSLRC 10 | 24.22 | 25.32 | 21.52 | 20.62 | 21.62 | 22.82 | 22.69 |
| 11. | PYPS-11 | PSLRC 4 x PSLRC 6 | 26.81 | 24.21 | 25.81 | 27.31 | 29.21 | 28.41 | 26.96 |
| 12. | PYPS-12 | PSLRC 4 x PSLRC 12 | 21.70 | 25.50 | 27.30 | 28.10 | 23.70 | 26.80 | 25.52 |
| 13. | PYPS-13 | PSLRC 2 x PSLRC 21 | 18.60 | 19.30 | 18.80 | 18.50 | 19.80 | 19.80 | 19.13 |
| 14. | PYPS-14 | PSLRC 20 x PSLRC 21 | 19.54 | 20.84 | 22.34 | 21.04 | 19.64 | 20.54 | 20.66 |
| 15. | PYPS-15 | PSLRC 2 x PSLRC 7 | 21.64 | 23.34 | 26.54 | 25.34 | 20.74 | 23.14 | 23.46 |
| 16. | PYPS-16 | PSLRC 20 x PSLRC 7 | 19.21 | 18.31 | 19.51 | 18.51 | 17.81 | 18.41 | 18.63 |
| 17. | PYPS-17 | PSLRC 2 x PSLRC 8 | 16.82 | 17.82 | 19.22 | 18.72 | 18.52 | 17.22 | 18.05 |
| 18. | PYPS-18 | PSLRC 3 x PSLRC 8 | 19.25 | 18.85 | 17.55 | 18.35 | 18.05 | 18.55 | 18.43 |
| 19. | PYPS-19 | PSLRC 4 x PSLRC 8 | 23.08 | 22.78 | 21.48 | 22.58 | 21.98 | 21.28 | 22.03 |
|  | CD |  | 0.87 | 0.87 | 0.87 | 0.87 | 0.87 | 0.87 | 0.87 |
|  | CV |  | 2.69 | 2.63 | 2.60 | 2.59 | 2.67 | 2.56 | 2.62 |
|  | General Mean: |  | 19.56 | 19.94 | 20.23 | 20.27 | 19.66 | 20.48 |  |

**SUPPLEMENTARY TABLE S5:** Bio-availability of Fe (%) in the grains of sorghum genotypes evolved from landraces during 2017- 2022

| **S. No.** | **Genotype** | **Pedigree** | **2017** | **2018** | **2019** | **2020** | **2021** | **2022** | **Mean** |
| --- | --- | --- | --- | --- | --- | --- | --- | --- | --- |
| 1. | PYPS-1 | PSLRC 2 x PSLRC 3 | 42.63 | 42.93 | 43.83 | 45.63 | 48.03 | 48.73 | 45.30 |
| 2. | PYPS-2 | PSLRC 2 x PSLRC 4 | 48.76 | 51.26 | 50.06 | 55.66 | 53.56 | 54.16 | 52.24 |
| 3. | PYPS-3 | PSLRC 3 x PSLRC 4 | 39.56 | 42.26 | 45.56 | 43.26 | 48.26 | 50.16 | 44.84 |
| 4. | PYPS-4 | PSLRC 2 x PSLRC 6 | 68.35 | 62.75 | 69.25 | 70.65 | 73.85 | 72.65 | 69.58 |
| 5. | PYPS-5 | PSLRC 21 x PSLRC 7 | 49.38 | 52.28 | 50.38 | 52.78 | 56.18 | 58.28 | 53.22 |
| 6. | PYPS-6 | PSLRC 3 x PSLRC 6 | 55.70 | 53.50 | 58.70 | 54.10 | 59.20 | 61.10 | 57.03 |
| 7. | PYPS-7 | PSLRC 3 x PSLRC 7 | 61.34 | 62.84 | 63.94 | 59.54 | 61.14 | 64.44 | 62.21 |
| 8. | PYPS-8 | PSLRC 8 x PSLRC 9 | 46.46 | 43.26 | 49.16 | 50.96 | 53.16 | 52.76 | 49.29 |
| 9. | PYPS-9 | PSLRC 8 x PSLRC 10 | 51.22 | 53.52 | 55.32 | 51.82 | 55.82 | 56.42 | 54.02 |
| 10. | PYPS-10 | PSLRC 9 x PSLRC 10 | 56.32 | 59.22 | 57.82 | 61.22 | 60.82 | 60.32 | 59.29 |
| 11. | PYPS-11 | PSLRC 4 x PSLRC 6 | 41.81 | 43.11 | 43.61 | 41.71 | 46.21 | 48.32 | 44.13 |
| 12. | PYPS-12 | PSLRC 4 x PSLRC 12 | 43.60 | 43.40 | 46.80 | 47.60 | 46.90 | 44.80 | 45.52 |
| 13. | PYPS-13 | PSLRC 2 x PSLRC 21 | 56.80 | 59.10 | 58.30 | 61.30 | 62.80 | 64.50 | 60.47 |
| 14. | PYPS-14 | PSLRC 20 x PSLRC 21 | 58.24 | 56.24 | 53.84 | 58.84 | 61.54 | 60.34 | 58.17 |
| 15. | PYPS-15 | PSLRC 2 x PSLRC 7 | 66.64 | 69.14 | 61.24 | 68.54 | 65.64 | 72.14 | 67.22 |
| 16. | PYPS-16 | PSLRC 20 x PSLRC 7 | 56.41 | 51.51 | 58.11 | 57.81 | 54.61 | 55.81 | 55.71 |
| 17. | PYPS-17 | PSLRC 2 x PSLRC 8 | 66.82 | 69.32 | 67.82 | 70.82 | 71.32 | 73.22 | 69.89 |
| 18. | PYPS-18 | PSLRC 3 x PSLRC 8 | 55.25 | 58.15 | 57.75 | 59.65 | 61.65 | 60.85 | 58.88 |
| 19. | PYPS-19 | PSLRC 4 x PSLRC 8 | 51.58 | 54.48 | 55.78 | 51.68 | 53.48 | 56.18 | 53.86 |
|  | CD |  | 0.84 | 0.84 | 0.84 | 0.84 | 0.84 | 0.84 | 0.84 |
|  | CV |  | 0.95 | 0.94 | 0.92 | 0.91 | 0.88 | 0.86 | 0.91 |
|  | General Mean: |  | 53.51 | 54.12 | 55.12 | 55.98 | 57.59 | 58.69 |  |

**SUPPLEMENTARY TABLE S6:** Zinc content (mg/100g) in the grains of sorghum genotypes evolved from landraces during 2017- 2022

| **S. No.** | **Genotype** | **Pedigree** | **2017** | **2018** | **2019** | **2020** | **2021** | **2022** | **Mean** |
| --- | --- | --- | --- | --- | --- | --- | --- | --- | --- |
| 1. | PYPS-1 | PSLRC 2 x PSLRC 3 | 5.93 | 6.73 | 7.53 | 8.23 | 7.93 | 7.83 | 7.36 |
| 2. | PYPS-2 | PSLRC 2 x PSLRC 4 | 8.46 | 7.16 | 7.76 | 8.76 | 8.36 | 8.16 | 8.11 |
| 3. | PYPS-3 | PSLRC 3 x PSLRC 4 | 4.76 | 5.56 | 5.16 | 5.96 | 6.26 | 5.66 | 5.56 |
| 4. | PYPS-4 | PSLRC 2 x PSLRC 6 | 7.65 | 7.25 | 7.55 | 7.05 | 7.75 | 7.45 | 7.45 |
| 5. | PYPS-5 | PSLRC 21 x PSLRC 7 | 4.78 | 5.48 | 4.58 | 5.88 | 5.58 | 5.18 | 5.25 |
| 6. | PYPS-6 | PSLRC 3 x PSLRC 6 | 6.40 | 6.10 | 6.70 | 6.30 | 6.70 | 5.90 | 6.35 |
| 7. | PYPS-7 | PSLRC 3 x PSLRC 7 | 4.84 | 5.64 | 5.74 | 5.84 | 4.94 | 5.34 | 5.39 |
| 8. | PYPS-8 | PSLRC 8 x PSLRC 9 | 7.86 | 6.76 | 7.56 | 8.16 | 7.76 | 7.46 | 7.59 |
| 9. | PYPS-9 | PSLRC 8 x PSLRC 10 | 7.22 | 7.52 | 7.42 | 6.52 | 6.32 | 6.82 | 6.97 |
| 10. | PYPS-10 | PSLRC 9 x PSLRC 10 | 4.32 | 4.82 | 5.52 | 5.82 | 6.02 | 5.42 | 5.32 |
| 11. | PYPS-11 | PSLRC 4 x PSLRC 6 | 4.21 | 5.11 | 5.91 | 5.51 | 4.61 | 4.81 | 5.03 |
| 12. | PYPS-12 | PSLRC 4 x PSLRC 12 | 5.00 | 4.60 | 5.50 | 5.80 | 5.60 | 5.20 | 5.28 |
| 13. | PYPS-13 | PSLRC 2 x PSLRC 21 | 8.20 | 7.80 | 6.40 | 7.00 | 7.70 | 7.90 | 7.50 |
| 14. | PYPS-14 | PSLRC 20 x PSLRC 21 | 6.54 | 6.94 | 6.04 | 6.64 | 7.04 | 6.24 | 6.57 |
| 15. | PYPS-15 | PSLRC 2 x PSLRC 7 | 7.64 | 6.84 | 7.44 | 7.04 | 7.34 | 7.24 | 7.26 |
| 16. | PYPS-16 | PSLRC 20 x PSLRC 7 | 5.41 | 6.21 | 6.51 | 5.81 | 5.61 | 6.01 | 5.93 |
| 17. | PYPS-17 | PSLRC 2 x PSLRC 8 | 7.22 | 7.52 | 7.72 | 7.92 | 6.82 | 7.32 | 7.42 |
| 18. | PYPS-18 | PSLRC 3 x PSLRC 8 | 7.05 | 7.85 | 7.65 | 6.45 | 6.95 | 7.55 | 7.25 |
| 19. | PYPS-19 | PSLRC 4 x PSLRC 8 | 6.28 | 6.78 | 7.28 | 7.48 | 6.88 | 7.08 | 6.96 |
|  | CD |  | 0.84 | 0.84 | 0.84 | 0.84 | 0.84 | 0.84 | 0.84 |
|  | CV |  | 8.07 | 7.88 | 7.68 | 7.54 | 7.66 | 7.76 | 7.76 |
|  | General Mean: |  | 6.30 | 6.45 | 6.63 | 6.74 | 6.64 | 6.55 |  |

**SUPPLEMENTARY TABLE S7:**Bio-availability of zinc (%) in the grains of sorghum genotypes evolved from landraces during 2017- 2022

| **S. No.** | **Genotype** | **Pedigree** | **2017** | **2018** | **2019** | **2020** | **2021** | **2022** | **Mean** |
| --- | --- | --- | --- | --- | --- | --- | --- | --- | --- |
| 1. | PYPS-1 | PSLRC 2 x PSLRC 3 | 51.33 | 53.83 | 50.63 | 52.53 | 54.53 | 52.93 | 52.63 |
| 2. | PYPS-2 | PSLRC 2 x PSLRC 4 | 43.76 | 46.66 | 45.76 | 48.06 | 48.56 | 49.36 | 47.03 |
| 3. | PYPS-3 | PSLRC 3 x PSLRC 4 | 64.46 | 68.26 | 69.56 | 67.86 | 65.26 | 72.76 | 68.03 |
| 4. | PYPS-4 | PSLRC 2 x PSLRC 6 | 50.55 | 53.45 | 55.75 | 51.85 | 57.55 | 56.45 | 54.27 |
| 5. | PYPS-5 | PSLRC 21 x PSLRC 7 | 45.18 | 43.58 | 46.88 | 48.28 | 47.78 | 50.18 | 46.98 |
| 6. | PYPS-6 | PSLRC 3 x PSLRC 6 | 43.30 | 46.60 | 45.50 | 49.50 | 46.90 | 48.00 | 46.63 |
| 7. | PYPS-7 | PSLRC 3 x PSLRC 7 | 51.44 | 53.64 | 56.74 | 68.34 | 61.84 | 60.34 | 58.72 |
| 8. | PYPS-8 | PSLRC 8 x PSLRC 9 | 41.76 | 43.56 | 45.26 | 46.56 | 41.86 | 44.46 | 43.91 |
| 9. | PYPS-9 | PSLRC 8 x PSLRC 10 | 61.82 | 60.32 | 66.72 | 64.32 | 68.82 | 65.62 | 64.60 |
| 10. | PYPS-10 | PSLRC 9 x PSLRC 10 | 55.42 | 51.72 | 56.82 | 53.92 | 59.72 | 62.32 | 56.65 |
| 11. | PYPS-11 | PSLRC 4 x PSLRC 6 | 60.61 | 61.81 | 67.51 | 72.31 | 71.81 | 69.21 | 67.21 |
| 12. | PYPS-12 | PSLRC 4 x PSLRC 12 | 48.20 | 49.30 | 53.50 | 51.70 | 55.30 | 52.40 | 51.73 |
| 13. | PYPS-13 | PSLRC 2 x PSLRC 21 | 50.90 | 52.50 | 57.80 | 61.30 | 60.60 | 58.50 | 56.93 |
| 14. | PYPS-14 | PSLRC 20 x PSLRC 21 | 69.34 | 63.54 | 69.04 | 65.64 | 61.84 | 66.84 | 66.04 |
| 15. | PYPS-15 | PSLRC 2 x PSLRC 7 | 44.44 | 48.14 | 43.64 | 49.44 | 50.74 | 51.54 | 47.99 |
| 16. | PYPS-16 | PSLRC 20 x PSLRC 7 | 51.31 | 54.61 | 55.51 | 56.21 | 58.61 | 59.81 | 56.01 |
| 17. | PYPS-17 | PSLRC 2 x PSLRC 8 | 41.22 | 50.62 | 49.32 | 48.52 | 44.82 | 46.32 | 46.80 |
| 18. | PYPS-18 | PSLRC 3 x PSLRC 8 | 45.65 | 49.55 | 49.05 | 45.75 | 50.85 | 52.45 | 48.88 |
| 19. | PYPS-19 | PSLRC 4 x PSLRC 8 | 46.18 | 48.48 | 51.68 | 50.28 | 53.58 | 50.78 | 50.16 |
|  | CD |  | 0.84 | 0.84 | 0.84 | 0.84 | 0.84 | 0.84 | 0.84 |
|  | CV |  | 1.00 | 0.96 | 0.93 | 0.92 | 0.91 | 0.90 | 0.94 |
|  | General Mean: |  | 50.89 | 52.64 | 54.56 | 55.39 | 55.84 | 56.33 |  |

**SUPPLEMENTARY TABLE S8:** Phenolics content (mg/g GAE) in the grains of sorghum genotypes evolved from landraces during 2017- 2022

| **S. No.** | **Genotype** | **Pedigree** | **2017** | **2018** | **2019** | **2020** | **2021** | **2022** | **Mean** |
| --- | --- | --- | --- | --- | --- | --- | --- | --- | --- |
| 1. | PYPS-1 | PSLRC 2 x PSLRC 3 | 60.83 | 61.63 | 63.53 | 65.73 | 67.83 | 66.43 | 64.33 |
| 2. | PYPS-2 | PSLRC 2 x PSLRC 4 | 70.27 | 71.77 | 76.77 | 73.67 | 77.47 | 75.27 | 74.20 |
| 3. | PYPS-3 | PSLRC 3 x PSLRC 4 | 40.27 | 41.77 | 45.27 | 46.27 | 43.47 | 44.07 | 43.51 |
| 4. | PYPS-4 | PSLRC 2 x PSLRC 6 | 61.75 | 65.85 | 66.45 | 68.25 | 69.05 | 69.35 | 66.78 |
| 5. | PYPS-5 | PSLRC 21 x PSLRC 7 | 42.50 | 43.59 | 45.80 | 44.20 | 47.50 | 48.40 | 45.31 |
| 6. | PYPS-6 | PSLRC 3 x PSLRC 6 | 50.00 | 53.30 | 55.45 | 51.70 | 51.50 | 52.50 | 52.41 |
| 7. | PYPS-7 | PSLRC 3 x PSLRC 7 | 51.24 | 50.74 | 51.54 | 56.64 | 49.84 | 58.34 | 53.06 |
| 8. | PYPS-8 | PSLRC 8 x PSLRC 9 | 65.76 | 66.66 | 65.26 | 67.86 | 64.56 | 66.16 | 66.04 |
| 9. | PYPS-9 | PSLRC 8 x PSLRC 10 | 61.32 | 66.02 | 61.82 | 63.52 | 65.72 | 68.12 | 64.42 |
| 10. | PYPS-10 | PSLRC 9 x PSLRC 10 | 59.52 | 62.52 | 58.72 | 63.92 | 68.10 | 70.52 | 63.90 |
| 11. | PYPS-11 | PSLRC 4 x PSLRC 6 | 43.22 | 48.42 | 47.62 | 49.52 | 45.72 | 46.22 | 46.78 |
| 12. | PYPS-12 | PSLRC 4 x PSLRC 12 | 50.71 | 51.61 | 53.91 | 55.81 | 56.61 | 58.31 | 54.48 |
| 13. | PYPS-13 | PSLRC 2 x PSLRC 21 | 70.30 | 71.80 | 76.20 | 75.70 | 76.30 | 77.80 | 74.68 |
| 14. | PYPS-14 | PSLRC 20 x PSLRC 21 | 73.14 | 70.44 | 73.54 | 72.84 | 69.04 | 72.64 | 71.94 |
| 15. | PYPS-15 | PSLRC 2 x PSLRC 7 | 75.64 | 71.44 | 78.24 | 70.74 | 72.44 | 73.04 | 73.59 |
| 16. | PYPS-16 | PSLRC 20 x PSLRC 7 | 51.61 | 52.31 | 56.81 | 59.31 | 55.91 | 57.41 | 55.56 |
| 17. | PYPS-17 | PSLRC 2 x PSLRC 8 | 61.82 | 62.62 | 65.72 | 66.82 | 63.52 | 66.32 | 64.47 |
| 18. | PYPS-18 | PSLRC 3 x PSLRC 8 | 77.25 | 79.55 | 78.95 | 81.55 | 84.35 | 85.65 | 81.22 |
| 19. | PYPS-19 | PSLRC 4 x PSLRC 8 | 42.60 | 43.40 | 47.60 | 49.70 | 48.00 | 48.30 | 46.58 |
|  | CD |  | 0.84 | 0.84 | 0.84 | 0.84 | 0.84 | 0.84 |  |
|  | CV |  | 0.87 | 0.85 | 0.82 | 0.81 | 0.82 | 0.80 |  |
|  | General Mean: |  | 58.40 | 59.75 | 61.53 | 62.30 | 61.94 | 63.41 |  |

**SUPPLEMENTARY TABLE S9:**Tannins (mg/g) in the grains of sorghum genotypes evolved from landraces during 2017- 2022

| **S. No.** | **Genotype** | **Pedigree** | **2017** | **2018** | **2019** | **2020** | **2021** | **2022** | **Mean** |
| --- | --- | --- | --- | --- | --- | --- | --- | --- | --- |
| 1. | PYPS-1 | PSLRC 2 x PSLRC 3 | 26.32 | 27.82 | 28.32 | 31.62 | 32.02 | 30.82 | 29.49 |
| 2. | PYPS-2 | PSLRC 2 x PSLRC 4 | 31.81 | 29.91 | 33.41 | 36.51 | 33.81 | 36.31 | 33.63 |
| 3. | PYPS-3 | PSLRC 3 x PSLRC 4 | 29.25 | 29.75 | 31.25 | 27.35 | 28.75 | 28.15 | 29.08 |
| 4. | PYPS-4 | PSLRC 2 x PSLRC 6 | 30.15 | 31.85 | 33.35 | 31.85 | 30.44 | 31.25 | 31.48 |
| 5. | PYPS-5 | PSLRC 21 x PSLRC 7 | 16.38 | 17.18 | 17.88 | 18.28 | 18.58 | 17.78 | 17.68 |
| 6. | PYPS-6 | PSLRC 3 x PSLRC 6 | 21.70 | 26.10 | 21.70 | 23.60 | 25.40 | 23.50 | 23.67 |
| 7. | PYPS-7 | PSLRC 3 x PSLRC 7 | 26.74 | 28.84 | 29.94 | 30.34 | 27.64 | 29.54 | 28.84 |
| 8. | PYPS-8 | PSLRC 8 x PSLRC 9 | 31.76 | 36.26 | 34.26 | 37.46 | 37.96 | 33.46 | 35.19 |
| 9. | PYPS-9 | PSLRC 8 x PSLRC 10 | 31.62 | 33.32 | 36.52 | 35.32 | 32.82 | 37.82 | 34.57 |
| 10. | PYPS-10 | PSLRC 9 x PSLRC 10 | 27.42 | 29.42 | 23.62 | 29.82 | 33.52 | 31.22 | 29.17 |
| 11. | PYPS-11 | PSLRC 4 x PSLRC 6 | 21.01 | 26.31 | 29.51 | 26.71 | 25.71 | 23.81 | 25.51 |
| 12. | PYPS-12 | PSLRC 4 x PSLRC 12 | 20.80 | 21.60 | 23.70 | 21.80 | 21.00 | 22.10 | 21.83 |
| 13. | PYPS-13 | PSLRC 2 x PSLRC 21 | 35.50 | 33.70 | 36.70 | 35.80 | 33.50 | 36.20 | 35.23 |
| 14. | PYPS-14 | PSLRC 20 x PSLRC 21 | 26.84 | 29.44 | 29.04 | 28.64 | 29.34 | 30.94 | 29.04 |
| 15. | PYPS-15 | PSLRC 2 x PSLRC 7 | 31.64 | 33.34 | 35.84 | 31.34 | 35.84 | 38.34 | 34.39 |
| 16. | PYPS-16 | PSLRC 20 x PSLRC 7 | 24.81 | 23.01 | 27.61 | 22.71 | 24.01 | 23.61 | 24.29 |
| 17. | PYPS-17 | PSLRC 2 x PSLRC 8 | 28.62 | 29.32 | 28.82 | 29.02 | 30.62 | 31.72 | 29.69 |
| 18. | PYPS-18 | PSLRC 3 x PSLRC 8 | 24.05 | 24.45 | 23.75 | 26.55 | 27.35 | 25.65 | 25.30 |
| 19. | PYPS-19 | PSLRC 4 x PSLRC 8 | 21.78 | 23.58 | 25.58 | 23.78 | 27.48 | 26.28 | 24.75 |
|  | CD |  | 0.87 | 0.87 | 0.87 | 0.87 | 0.87 | 0.87 | 0.87 |
|  | CV |  | 1.96 | 1.86 | 1.81 | 1.82 | 1.80 | 1.79 | 0.81 |
|  | General Mean: |  | 26.75 | 28.17 | 28.99 | 28.87 | 29.25 | 29.35 |  |

**SUPPLEMENTARY TABLE S10:**Anti-oxidant activity (%) in the grains of sorghum genotypes evolved from landraces during 2017- 2022

| **S. No.** | **Genotype** | **Pedigree** | **2017** | **2018** | **2019** | **2020** | **2021** | **2022** | **Mean** |
| --- | --- | --- | --- | --- | --- | --- | --- | --- | --- |
| 1. | PYPS-1 | PSLRC 2 x PSLRC 3 | 73.82 | 75.62 | 77.82 | 76.32 | 75.92 | 79.32 | 76.47 |
| 2. | PYPS-2 | PSLRC 2 x PSLRC 4 | 84.61 | 81.01 | 83.21 | 81.81 | 86.61 | 83.41 | 83.44 |
| 3. | PYPS-3 | PSLRC 3 x PSLRC 4 | 41.25 | 46.35 | 47.55 | 44.45 | 41.85 | 43.55 | 44.17 |
| 4. | PYPS-4 | PSLRC 2 x PSLRC 6 | 71.75 | 68.35 | 69.55 | 71.35 | 68.85 | 70.65 | 70.08 |
| 5. | PYPS-5 | PSLRC 21 x PSLRC 7 | 50.28 | 51.48 | 53.58 | 51.88 | 50.88 | 52.78 | 51.81 |
| 6. | PYPS-6 | PSLRC 3 x PSLRC 6 | 51.20 | 56.50 | 51.40 | 53.80 | 55.30 | 56.10 | 54.05 |
| 7. | PYPS-7 | PSLRC 3 x PSLRC 7 | 43.64 | 45.84 | 46.74 | 47.54 | 49.34 | 48.84 | 46.99 |
| 8. | PYPS-8 | PSLRC 8 x PSLRC 9 | 73.36 | 76.76 | 79.46 | 73.56 | 75.66 | 76.16 | 75.83 |
| 9. | PYPS-9 | PSLRC 8 x PSLRC 10 | 68.32 | 67.62 | 69.52 | 63.92 | 68.82 | 71.52 | 68.29 |
| 10. | PYPS-10 | PSLRC 9 x PSLRC 10 | 64.82 | 69.32 | 69.02 | 68.62 | 71.02 | 74.82 | 69.60 |
| 11. | PYPS-11 | PSLRC 4 x PSLRC 6 | 52.01 | 56.41 | 55.81 | 51.81 | 51.61 | 53.11 | 53.46 |
| 12. | PYPS-12 | PSLRC 4 x PSLRC 12 | 43.20 | 45.50 | 40.80 | 47.60 | 48.30 | 46.60 | 45.33 |
| 13. | PYPS-13 | PSLRC 2 x PSLRC 21 | 75.30 | 72.40 | 70.20 | 72.00 | 69.60 | 70.30 | 71.63 |
| 14. | PYPS-14 | PSLRC 20 x PSLRC 21 | 71.44 | 76.34 | 71.84 | 70.34 | 70.74 | 72.34 | 72.17 |
| 15. | PYPS-15 | PSLRC 2 x PSLRC 7 | 66.34 | 68.74 | 68.84 | 68.64 | 72.04 | 70.84 | 69.24 |
| 16. | PYPS-16 | PSLRC 20 x PSLRC 7 | 61.81 | 63.51 | 65.61 | 66.81 | 67.01 | 67.21 | 65.33 |
| 17. | PYPS-17 | PSLRC 2 x PSLRC 8 | 69.32 | 64.32 | 68.02 | 66.52 | 69.02 | 68.82 | 67.67 |
| 18. | PYPS-18 | PSLRC 3 x PSLRC 8 | 51.85 | 53.35 | 58.55 | 56.25 | 51.35 | 54.25 | 54.27 |
| 19. | PYPS-19 | PSLRC 4 x PSLRC 8 | 81.28 | 80.48 | 83.68 | 85.38 | 86.68 | 89.78 | 84.55 |
|  | CD |  | 0.87 | 0.87 | 0.87 | 0.87 | 0.87 | 0.87 | 0.87 |
|  | CV |  | 0.83 | 0.82 | 0.81 | 0.82 | 0.81 | 0.80 | 0.81 |
|  | General Mean: |  | 62.92 | 64.20 | 64.80 | 64.14 | 64.77 | 65.81 |  |
